# Fusing conveyor-belt images with existing in-line weight records for flue-cured tobacco leaf grading: a strict date-held-out evaluation

**DOI:** 10.3389/fpls.2026.1970460

**Published:** 2026-10-26

**Authors:** Qi Xiong, Wuke Li, Wenxue Tan

**Affiliations:** 1 School of Computer and Electrical Engineering, Hunan University of Arts and Science, Changde, China; 2 Key Laboratory of Hunan Province for Control Technology of Distributed Electric Propulsion Air Vehicle, Changde, China

**Keywords:** agricultural computer vision, cluster bootstrap, cross-batch evaluation, data leakage, flue-cured tobacco, in-line weighing, leaf grading, multimodal fusion

## Abstract

Deep learning models for flue-cured tobacco leaf grading are often evaluated using randomly split datasets, which can place images from the same acquisition day in both training and test sets. This may overestimate performance because batch, lighting, and moisture conditions are shared across the splits. This study examined whether existing in-line weight records can improve image-based grading under a stricter, date-grouped evaluation protocol. We used 25,325 paired conveyor-belt images and weight records collected on 34 acquisition dates during the 2025 season. Acquisition dates were kept separate across the training, validation, and test sets. We compared image-only, weight-only, and four image–weight fusion models. A ResNet-50 classifier achieved 86.2% top-1 accuracy with random splitting but only 43.4% when whole acquisition dates were held out, showing that random splits substantially overstate real-world performance. Weight alone performed poorly and varied strongly across batches. Combining weight with images produced small gains of about 1–2 percentage points; among the four fusion architectures of the main comparison, only late fusion yielded a statistically significant improvement over the image-only model, and two deeper fusion baselines (FiLM feature conditioning and cross-modal attention) added in a follow-up experiment did not exceed it either. Scalar gating for adaptively weighting the two modalities did not reliably identify image-model failures. These results indicate that grading studies should report how data are split, disclose date-level groupings, and distinguish genuine generalization from mere random-seed stability. Overall, in-line weight records provide limited but useful complementary information without requiring additional hardware. Date-grouped evaluation is essential for assessing whether tobacco grading models can generalize across production batches.

## 1 Introduction

Flue-cured tobacco is a major cash crop in many growing regions. The grade assigned to each leaf at the purchasing station determines its price, which directly affects farm income (Standardization Administration of China, 1992; Li et al., 2026). Grading follows the national standard GB 2635–1992 (Standardization Administration of China, 1992), which defines 42 grades by leaf position group (X: lugs, C: cutters, B: leaf) and quality factors (color, maturity, oil, body, damage). As a recent review of tobacco classification technologies emphasizes, grading is the vital bridge linking raw leaf properties to end-product quality, and inconsistent grading propagates directly into blending losses and price disputes downstream (Li et al., 2026). The task is performed visually by certified raters and is widely recognized as subjective, fatiguing, and difficult to standardize across stations and seasons (Zhang and Zhang, 2011; Li et al., 2026).

Automating this task has a long research history. Early work relied on conventional image processing and hand-crafted features: color- and texture-based fuzzy evaluation (Zhang and Zhang, 2011), machine-vision pipelines for harvesting-stage classification (Guru et al., 2012), color-and quality-based grading rules (Harjoko et al., 2019), and Haar-cascade leaf detection as a preprocessing step (Marzan and Marcos, 2018). These methods established that leaf appearance carries grade-relevant signal, but their reliance on hand-tuned thresholds limited robustness across origins and lighting conditions.

Deep learning has been repeatedly applied to this problem, with reported accuracies ranging from roughly 80% to above 99% (Lu et al., 2022; Chen et al., 2023; He et al., 2023; Li et al., 2023; Lu et al., 2023; Xin et al., 2023; Chen et al., 2024; Pang et al., 2026). Representative examples include densely connected convolutional networks for large-scale grading (Xin et al., 2023), hierarchical feature-fusion architectures specifically designed to distinguish between position-group and quality-number cues (Pang et al., 2026), cross-modal enhancement networks that integrate leaf images with auxiliary descriptors (He et al., 2023), end-to-end deep classifiers deployed on grading lines (Li et al., 2023), bilinear and improved bilinear CNNs that model fine-grained inter-grade differences (Lu et al., 2022; Lu et al., 2023), dual-encoder feature-reinforcement networks (Chen et al., 2023), and vein-information-guided fine-grained classifiers that precisely address the adjacent-grade confusions purchasing stations encounter (Chen et al., 2024). Complementary efforts have pursued spectral hardware rather than RGB imaging alone, including near-infrared and hyperspectral acquisition combined with extreme learning machines or CNNs (Bin et al., 2017; Wei et al., 2024; Zhang et al., 2024) and transmission imaging (He et al., 2023) to expose internal leaf structure invisible to surface photography.

Two observations motivate the present study. First, nearly all of the accuracies above are computed under random image-level train/test splits. Because acquisition is organized in sessions (a station, a date, a camera state, often a single origin and cultivar), random splits place near-duplicate acquisition conditions in both partitions, and a model can succeed by learning session-specific appearance rather than grade-relevant morphology. This is not a hypothetical concern: in our own data, the same fully fine-tuned ResNet-50 reaches 86.2% under a random split but only 43.4% when whole acquisition dates are held out (Section 4.1)—a 42.8-point gap attributable to partition design alone. Similar laboratory-to-field collapses, in which evaluation design rather than backbone choice explains most of the reported gap, have been extensively documented in plant disease recognition (Ferentinos, 2018; Fenu and Malloci, 2022; Ahmad et al., 2023; Tian et al., 2024), and group-aware, session-held-out evaluation has been proposed there as the remedy. Reported tobacco-grading splits are, to our knowledge, never constructed this way; for example, one recent large-scale study (Pang et al., 2026) evaluates on a single random partition of over 200,000 images without discussing acquisition-session structure. Second, some studies push accuracy further by using specialized hardware, such as transmission imaging (He et al., 2023) or near-infrared and hyperspectral cameras (Bin et al., 2017; Wei et al., 2024; Zhang et al., 2024). This limits practical adoption, even when the accuracy gains reported under random splits are genuine.

This paper explores an overlooked alternative: purchasing lines already weigh every single leaf on an in-line electronic scale for settlement. This weight record is free at the margin—it requires no additional sensing hardware—and it is physically informative, because leaf weight correlates with position group and quality grade (Marzan and Ruiz, 2019; Singh et al., 2024), while being insensitive to illumination and camera drift. Multimodal sensor fusion has delivered measurable gains elsewhere in agricultural quality assessment, for instance combining RGB with thermal imaging for fruit quality grading (Ali et al., 2023) and fusing multiple sensing modalities for fruit maturity assessment (Liu et al., 2024), which suggests conveyor images and weight records could be complementary rather than redundant for tobacco. We ask three questions: (i) How is fine tobacco grading best evaluated so that reported accuracy reflects cross-batch generalization? (ii) Do existing weight records add measurable value to conveyor-belt images under such an evaluation? (iii) Which fusion strategy actually helps? Can a learned gate down-weight the image stream when it is unreliable? Here, the gate is a sample-specific coefficient that controls how much the image and weight modalities each contribute.

Our answers are deliberately conservative. Under a strict, programmatically verified date-held-out protocol, fusion gains over the image modality are small (1–2 points) and only late fusion is statistically robust; the weight signal itself is strongly batch-dependent; and a scalar gate does not learn to detect visual failure. We consider these honest negatives and boundary results a contribution in their own right, and provide the complete date-level fold assignments in **Supplementary Appendix A** to support verification of the evaluation protocol. The main contributions of this study are as follows:

1. We establish a strict date-held-out evaluation protocol for tobacco grading to assess generalization across production days rather than performance under conventional random splitting. Each of the 34 acquisition dates is treated as an indivisible group, with train/validation/test date separation programmatically verified; grades that cannot satisfy this constraint are excluded from the primary evaluation and reported separately.
2. To our knowledge, this is the first study to fuse existing in-line weight data with conveyor-belt images for fine-grained nine-grade tobacco grading, without requiring additional sensing hardware (Bin et al., 2017; He et al., 2023; Wei et al., 2024; Zhang et al., 2024).
3. Under the same data splits and random seeds, we compare four model designs: feature concatenation, gated fusion, late-score averaging, and hierarchical classification. Statistical uncertainty is assessed using cluster bootstrapping at the acquisition-date level.

## 2 Related work

### 2.1 Computer vision for tobacco leaf grading

Early systems used color, shape, and texture features with classical classifiers: fuzzy comprehensive evaluation on color and texture descriptors (Zhang and Zhang, 2011), machine-vision pipelines targeting automatic harvesting rather than purchasing-line grading (Guru et al., 2012), rule-based color-and-quality grading (Harjoko et al., 2019), template-matching and Haar-cascade leaf detection as a necessary preprocessing step before classification (Marzan and Marcos, 2018), and CNN-based classification built directly on top of such image-processing pipelines (Marzan and Ruiz, 2019). These studies demonstrated that leaf appearance is informative but exposed the fragility of hand-crafted features under uncontrolled illumination and background. End-to-end deep convolutional pipelines subsequently removed the hand-crafted stage altogether (Dasari and Prasad, 2019).

Deep networks then substantially improved reported accuracy. Representative lines of work include densely connected networks for large-scale grading (Xin et al., 2023), hierarchical feature fusion explicitly separating position-group and quality-number cues and reporting 99.95% on 201,418 images and 24 grades (Pang et al., 2026), an end-to-end deep classification system evaluated directly on a grading line (Li et al., 2023), bilinear and improved bilinear CNNs for fine-grained inter-grade discrimination (Lu et al., 2022; Lu et al., 2023), vein-guided fine-grained classification targeting adjacent-grade confusion (Chen et al., 2024), and feature-reinforced dual-encoder aggregation (Chen et al., 2023). A recent minireview situates all of this work within the broader chain from raw leaf properties to end-product quality, underscoring that grading errors propagate downstream into blending and pricing (Li et al., 2026). Spectral modalities extend beyond RGB: NIR with extreme learning machines (Bin et al., 2017), visible–NIR fusion (Zhang et al., 2024), and hyperspectral imaging of folded leaves (Wei et al., 2024). CMENet (He et al., 2023) fuses surface and transmission images with a cross-modal enhancement network, reporting 80.15% — the closest multimodal predecessor to the present study — but requires dedicated transmission-imaging hardware. A parallel stream targets maturity rather than station grading: CNN-based maturity identification with lightweight backbones for in-field use (Zhang et al., 2023; Wu et al., 2024), maturity discrimination on mobile devices (Chen et al., 2023), and tobacco-shred variety classification (Niu et al., 2022). Spectral efforts further include rapid NIR hyperspectral grading of flue-cured tobacco (Zou et al., 2026), hand-held NIR spectrometers coupled with PSO-optimized extreme learning machines (Li et al., 2020), and proximal hyperspectral maturity classification (Wu et al., 2024).

Across this literature, two gaps remain unaddressed. First, with the exception of CMENet (He et al., 2023), virtually all inputs are single-modality RGB, and to our knowledge no prior work uses the per-leaf weight records that purchasing lines already produce as a byproduct of settlement. Second, reported splits are image-level random or left unspecified, so cross-batch generalization is essentially unknown (Pang et al., 2026);, for example, evaluates on a single random partition of its 201,418 images without discussing acquisition-session structure, and none of (Lu et al., 2022; Chen et al., 2023; Li et al., 2023; Lu et al., 2023; Xin et al., 2023; Chen et al., 2024; Wei et al., 2024) report session- or date-held-out results.

### 2.2 Multimodal fusion in agricultural quality assessment

Sensor fusion surveys for fruit and vegetable quality (Singh et al., 2024) document consistent gains from combining RGB with thermal (Ali et al., 2023), spectral, or depth streams, using early (feature concatenation), late (decision averaging), and intermediate fusion strategies. Concrete instances include RGB–thermal fusion for pineapple quality determination (Ali et al., 2023) and multi-sensor fusion for tomato maturity assessment (Liu et al., 2024), both reporting improvements over any single modality alone. In tobacco specifically, feature-level fusion has been used for curing-stage recognition with a Bayesian mixture-of-experts model (Zhao et al., 2025). Learned gating — a scalar or vector coefficient that reweights modality embeddings according to estimate per-sample reliability — is a common intermediate strategy in this literature, motivated by the intuition that different sensing channels degrade under different conditions.

However, fusion in this body of work is almost universally evaluated under the same random-split regime as unimodal studies, and modality reliability is rarely analyzed as a function of acquisition batch or session. Our results suggest this omission matters in practice, not just in principle: the reliability of each modality in our data varies strongly and unpredictably across acquisition dates, and a simple scalar gate — the mechanism most fusion studies rely on — fails to detect when the dominant modality (vision) is unreliable. This points to a broader limitation of applying agricultural sensor-fusion recipes developed under random splits directly to purchasing-line or field deployment settings.

### 2.3 Evaluation pitfalls in agricultural computer vision

Laboratory-to-field performance collapse is well documented in plant disease recognition (Ferentinos, 2018; Fenu and Malloci, 2022; Ahmad et al., 2023; Tian et al., 2024): models evaluated under field-realistic conditions lose large fractions of their nominal accuracy, and evaluation design, not backbone choice, explains much of the gap. For example (Fenu and Malloci, 2022), shows that laboratory-collected and field-collected datasets for the same disease classification task yield substantially different apparent performance for identical models (Ahmad et al., 2023), traces generalization failure specifically to controlled-versus-field acquisition conditions, and (Tian et al., 2024) argues that crop-specific evaluation protocols, not merely larger models, are needed to make reported accuracy practically meaningful. Group-aware evaluation — plant-level, field-level, or session-level held-out sets — is the consistently recommended remedy across this literature. Field-robustness studies in adjacent tobacco-vision tasks point in the same direction, for example diffusion-augmented disease identification and grading under low-resolution complex scenes (Xiong et al., 2024).

Tobacco grading inherits all of these risks and adds one specific to purchasing-line operation: the acquisition session is simultaneously the business batch, so grade distributions, lighting, and even scale calibration shift together with origin and date. This coupling means that a random split does not merely leak generic visual similarity (as in disease recognition) but can also leak batch-level label-distribution information, likely amplifying the apparent-versus-real accuracy gap relative to other agricultural vision tasks. Our protocol is a direct application of group-aware evaluation to purchasing-line grading, with acquisition dates as the atomic, non-splittable group — to our knowledge the first time this remedy has been applied to tobacco grading specifically.

## 3 Materials and methods

### 3.1 Data acquisition and image–weight pairing

Data were collected on a commercial flue-cured tobacco purchasing line during the 2025 purchasing season. Each leaf passes a conveyor camera (RGB image) and an in-line electronic scale (per-leaf weight in grams) whose readings are logged with timestamps. Grade labels were assigned on-site by certified raters at the purchasing stations following GB 2635–1992 (Standardization Administration of China, 1992) as part of routine operation; labels were not re-annotated for this study (see Section 3.6 for a transparent discussion of annotation and measurement metadata).

The raw pool contains 26,105 conveyor images. Pairing images with weight logs by timestamp yields 25,420 pairs (685 images had no unique weight record). Removing 70 records with non-positive weight (scale faults) and 25 samples of the non-standard grades BK1/BK2 leaves 25,325 pairs covering 11 grades (**Table 1**). The 34 distinct acquisition dates come from three named stations (Zhulin, Muyang, DP) plus a subset whose station metadata was not recorded in the purchased dataset. Because every date is effectively single-station in the logs, the acquisition date serves as the atomic batch unit (Section 3.2). Recorded cultivars are Yun87, Yun97, and Yn223, with cultivar metadata missing for part of the collection. Within the B and C position groups, grade-wise mean weight decreases monotonically as the quality number increases (from 1F toward 3F/4F). The X group shows no stable monotone relation. This instability may reflect batch composition rather than physiology, as its two finer grades (X3F, X4F) each come from only one or two acquisition dates. The acquisition system also logs a per-leaf length field whose physical unit and measurement procedure could not be verified; we therefore exclude it from all quantitative analysis rather than build conclusions on an unverifiable quantity. From the data files themselves we can further establish that the images are PNG frames of approximately 2320–2384 × 980–1000 pixels (four distinct frame sizes, suggesting multiple camera units), that scale readings are recorded with 0.01 g resolution, and that event timestamps have one-second resolution. The camera model, scale precision class and calibration cycle, and the number of raters were not provided by the data vendor and are listed among the limitations. The per-date grade composition is visualized in **Figure 1** and listed in full in **Supplementary Appendix A** (**Supplementary Table A2**); the 21 dates whose station field is ‘unknown’ are analyzed separately there.

**Table 1:** Data flow and grade statistics.

Grade	Position	Samples	Dates	Weight (g), mean ± std
B1F	B	1938	10	27.86 ± 7.97
B2F	B	2285	10	22.43 ± 6.35
B3F	B	2289	11	15.25 ± 5.17
C1F	C	1827	9	34.28 ± 8.17
C2F	C	2901	16	25.11 ± 6.77
C3F	C	5765	17	20.47 ± 7.35
C3L	C	1412	14	17.86 ± 5.98
C4F	C	2474	12	11.48 ± 4.61
X2F	X	3381	13	17.33 ± 6.04
X3F	X	346	1	13.04 ± 3.38
X4F	X	707	2	15.64 ± 5.72
**Total**	**—**	**25325**	**34**	**20.59 ± 8.80**

Dataset curation workflow: 26,105 raw conveyor images were collected. Timestamp-based pairing with in-line scale records yielded 25,420 image–weight pairs, with 685 images excluded because no unique weight record was available. After removing 70 scale-fault records with non-positive weights and 25 non-standard BK1/BK2 samples, 25,325 valid pairs covering 11 grades remained. The main 9-grade experiment used 24,272 pairs after excluding X3F and X4F (1,053 samples), because these two rare grades lacked sufficient acquisition dates for strict date-held-out evaluation and were therefore evaluated separately in the supplementary experiment (Section 3.2).

Bold values indicate the column totals.

**Figure 1:**
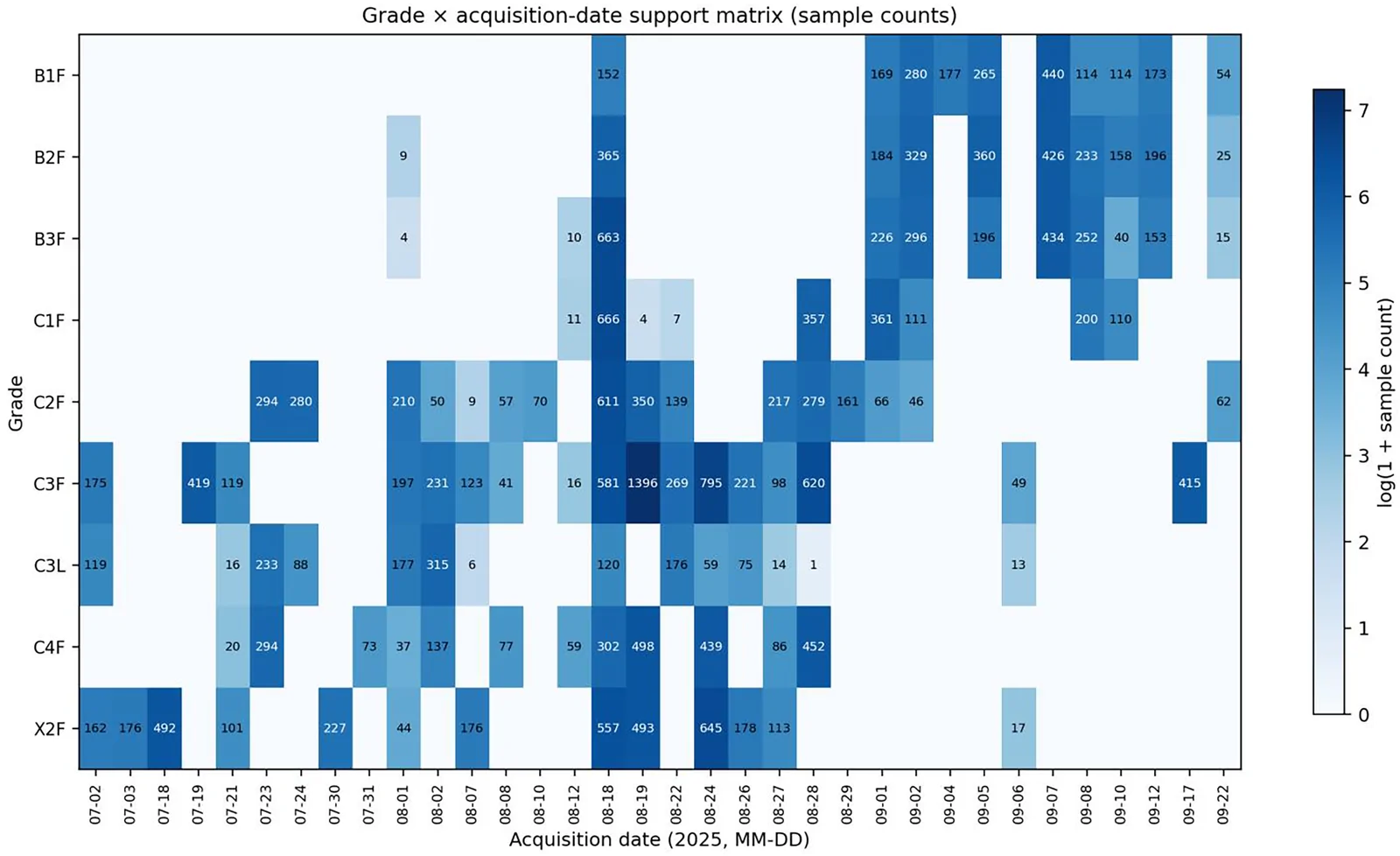
Grade × acquisition-date support matrix (sample counts, nine main grades, 34 dates).

Images were cropped to a fixed leaf region of interest and resized to 448 × 224 pixels; all experiments below use these cached crops. **Figure 2** shows the dataset composition, per-grade weight distributions, and the acquisition calendar.

**Figure 2:**
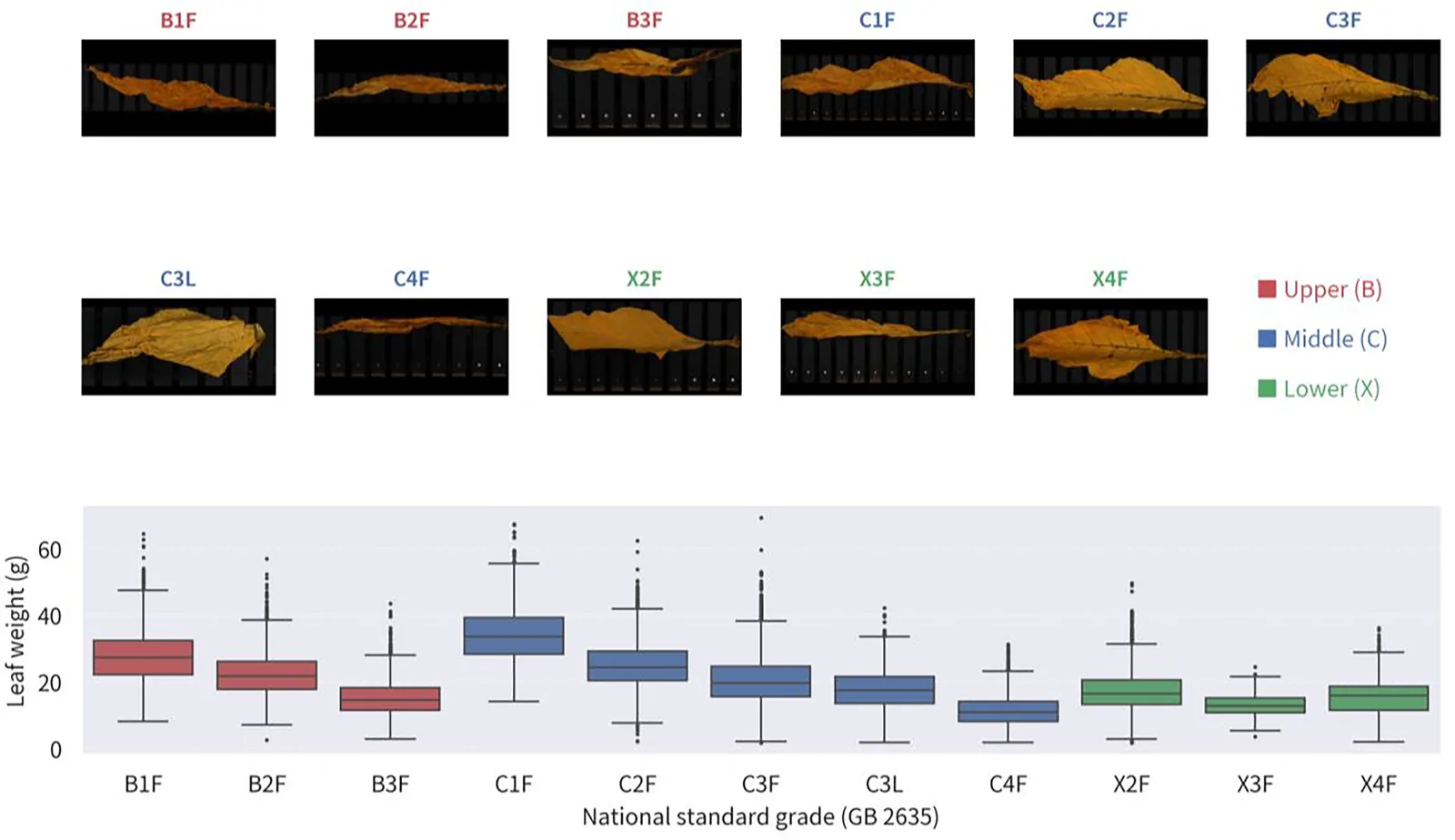
summarizes representative leaf images and the per-grade weight distributions.

### 3.2 Strict date-held-out evaluation protocol

The protocol has three rules. (1) Atomic groups. The 34 acquisition dates are indivisible across the 3-fold rotation: in every fold, each date is assigned as a whole to exactly one of training, validation, or test. A programmatic assertion verifies that the three partitions share zero dates in every fold. This closes not only the obvious within-grade leakage but also the subtler cross-grade leakage in which the same date appears in training for one grade and in test for another. (2) Grade coverage. Dates are partitioned into three buckets (12/11/11 dates; ≈8,000 samples each) balanced so that every bucket contains every grade with at least 442 samples; the buckets rotate through test/validation/training roles, so every date is tested exactly once. (3) Rare grades. Grades with fewer than three dates cannot participate in any strictly date-held-out partition. Our main experiment therefore covers the nine grades with ≥3 dates (24,272 pairs). X3F (1 date) and X4F (2 dates) are evaluated only in a supplementary 11-grade experiment, in which their fallback splits-single-date stratified random for X3F and two-date fixed split for X4F- are explicitly flagged per sample in the released split files rather than hidden inside the aggregate metric. The complete assignment of the 34 dates to partitions in each fold is listed in **Supplementary Appendix A** (**Supplementary Table A1**).

### 3.3 Models

The neural architectures are built upon two modality-specific encoders. For the image modality, all image-based models use a ResNet-50 (He et al., 2016) backbone initialized with ImageNet weights (Deng et al., 2009). Layers conv1–layer3 are frozen, whereas layer4 is fine-tuned, and global average pooling produces a 2048-D image feature *f*. in each fold using the mean and standard deviation computed from the training partition only and is then encoded by a two-layer MLP (1–64–64, ReLU) to produce a 64-D weight embedding *h_w_*. Based on these modality-specific representations, we construct four neural architectures: two unimodal baselines (vision-only and weight-only) and two jointly trained feature-fusion models (concatenation and gated fusion). The unimodal baselines quantify the predictive contribution of each modality separately, whereas the fusion models evaluate whether image and weight information provide complementary cues. The four architectures are illustrated in **Figure 3** and described below.

**Figure 3:**
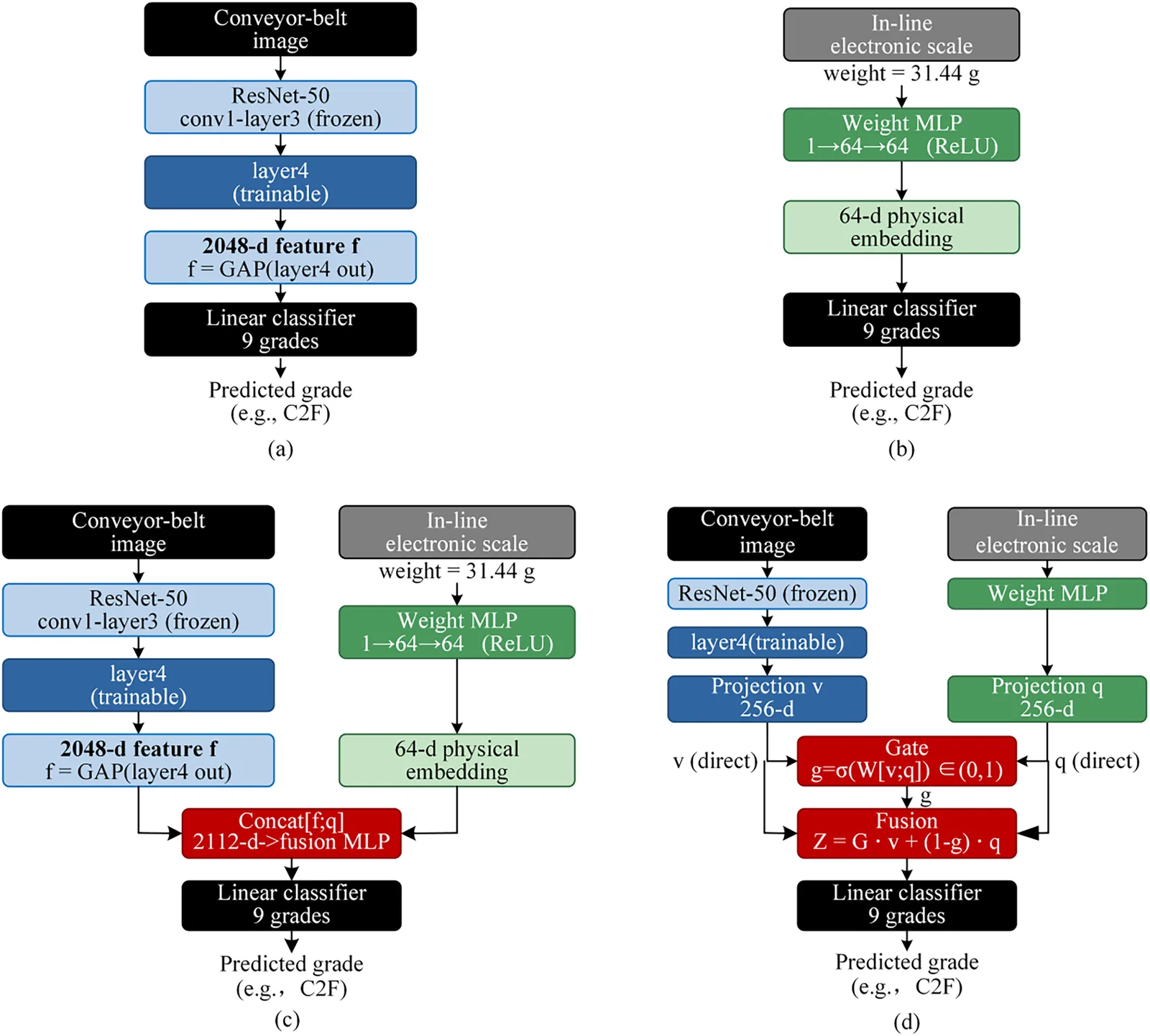
Architectures of the two unimodal neural baselines and the two jointly trained feature-fusion models. **(a)** vision-only. **(b)** weight-only. **(c)** concatenation fusion. **(d)** gated fusion. The image branch uses a ResNet-50 backbone with conv1–layer3 frozen and layer4 fine-tuned, followed by global average pooling. The scalar leaf weight is standardized using training-fold statistics and encoded by a two-layer MLP. In concatenation fusion, the projected image and weight embeddings are concatenated and passed through a fusion MLP. In gated fusion, a learned scalar gate combines the two 256-D projections before classification. All models predict the nine grades included in the main experiment.

vision-only: the 2048-D image feature f is passed directly to a linear nine-class classifier.

weight-only: the 64-D weight embedding *h_w_* is passed to a linear nine-class classifier.

concatenation fusion: the 2048-D image feature f and the 64-D weight embedding h_w are concatenated into a 2112-D vector, which is passed through a fusion layer (Linear 2112→256 followed by ReLU) and then a linear nine-class classifier.

gated fusion: Gated fusion learns a scalar mixing coefficient that decides, for each sample, how much the image stream and the weight stream should contribute. The image and weight embeddings are first projected into a shared 256-D space; their projected representations, *v* and *q*, are then concatenated and fed through a sigmoid unit to produce:


$$g=\sigma (w^{T}[v;q])$$

where *g* is the scalar gate. The fused representation is then computed as:


z=gv+(1−g)q

Thus, values of g closer to 1 assign greater weight to the image representation, whereas values closer to 0 favor the weight representation; g = 0.5 gives the two projected representations equal coefficients. The gate is learned jointly with the classifier and is not supervised using a separate modality-reliability target.

For completeness, every projection above is a single linear layer followed by a ReLU (v = ReLU(W_v f) with W_v ∈ R^{256×2048}; q = ReLU(W_q h_w) with W_q ∈ R^{256×64}; all dimensionalities are listed in **Supplementary Appendix E**, **Supplementary Table E2**), and the gate is a single Linear (512→1) layer with a sigmoid. A full symbol and dimensionality specification is given in **Supplementary Appendix E** (**Supplementary Tables E1, E2**).

Two additional multimodal strategies, Late probability averaging (Late) and Hierarchical classification (Hier), are evaluated as alternatives to the jointly trained feature-fusion architectures shown in **Figure 4**. Late combines the class-probability vectors produced by independently trained vision-only and weight-only models by unweighted averaging and introduces no additional trainable parameters. Hier first predicts the leaf-position group (B/C/X) and then predicts the grade within the selected group. Because the main experiment contains only X2F in the X group, the X branch is resolved directly. Both hierarchical stages are implemented as small MLPs operating on the frozen image embedding and the standardized weight scalar. **Figure 4** provides a strategy-level comparison of the four multimodal integration methods.

**Figure 4:**
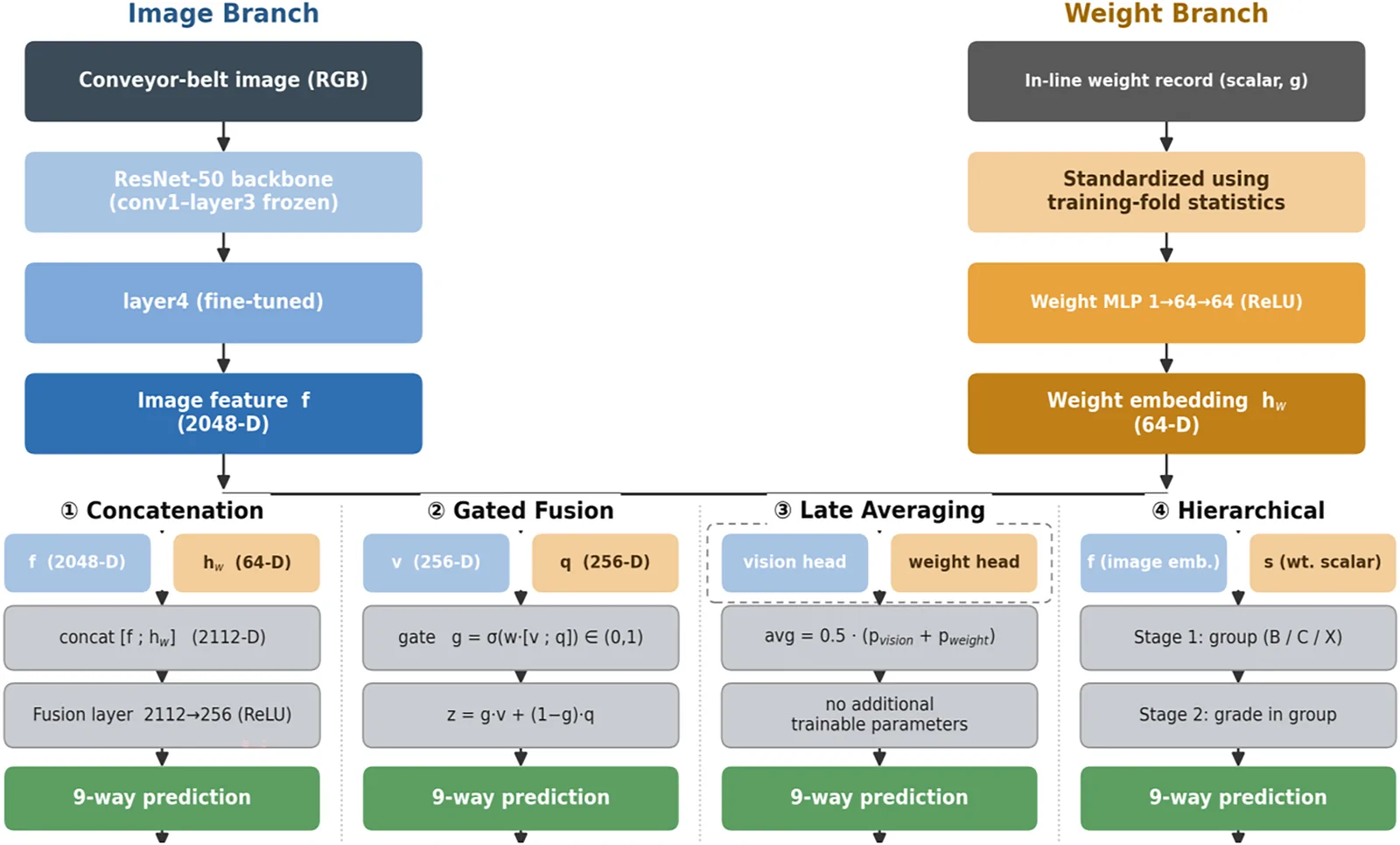
Strategy-level comparison of the four multimodal integration methods. Concatenation and gated fusion combine image and weight representations at the feature level and are trained jointly. Late averaging combines the class-probability vectors of independently trained unimodal models by simple averaging, introducing no additional trainable parameters. Hierarchical classification first predicts the position group (B/C/X) and then predicts the grade within the selected group. The main experiment includes B1F, B2F, B3F, C1F, C2F, C3F, C3L, C4F, and X2F.

To isolate the predictive contribution of the weight signal, we additionally evaluate the following classical weight-only baselines on the identical folds: multinomial logistic regression, RBF-SVM, histogram-based gradient boosting, Gaussian naive Bayes, ordinal logistic regression, and a one-dimensional threshold classifier whose boundaries are placed at the midpoints between sorted per-grade training means.

All neural models are trained using AdamW, with a learning rate of 1×10−4 for the trainable backbone stage and 1×10−3 for newly initialized heads, and a weight decay of 1×10−4. We use a cosine learning-rate schedule with one epoch of warm-up, label smoothing of 0.05, exponential moving averaging with a decay of 0.995, and mixed-precision training. Early stopping is based on validation accuracy with a patience of four epochs and a maximum of 12 epochs. The batch size is 32. Each neural configuration is repeated using three random seeds (42, 7, and 2025) in every fold.

### 3.4 Metrics

We report top-1 accuracy, balanced accuracy, macro-F1, quadratic weighted kappa (QWK) on an ordinal encoding (position base + quality number), ordinal MAE, and an adjacent-tolerance accuracy reflecting purchasing practice, in which a prediction is accepted if it shares the true grade’s position letter and color suffix and differs by at most one quality number (e.g., B2F ↔ B1F/B3F is tolerated; C3F ↔ C3L is not, since color differs). **Figure 5** shows the resulting 9×9 adjacency-tolerance matrix. Summing the tolerated cells per row (row totals shown on the right) gives a mean chance level of 19/81 ≈ 23.5% under uniform random guessing.

**Figure 5:**
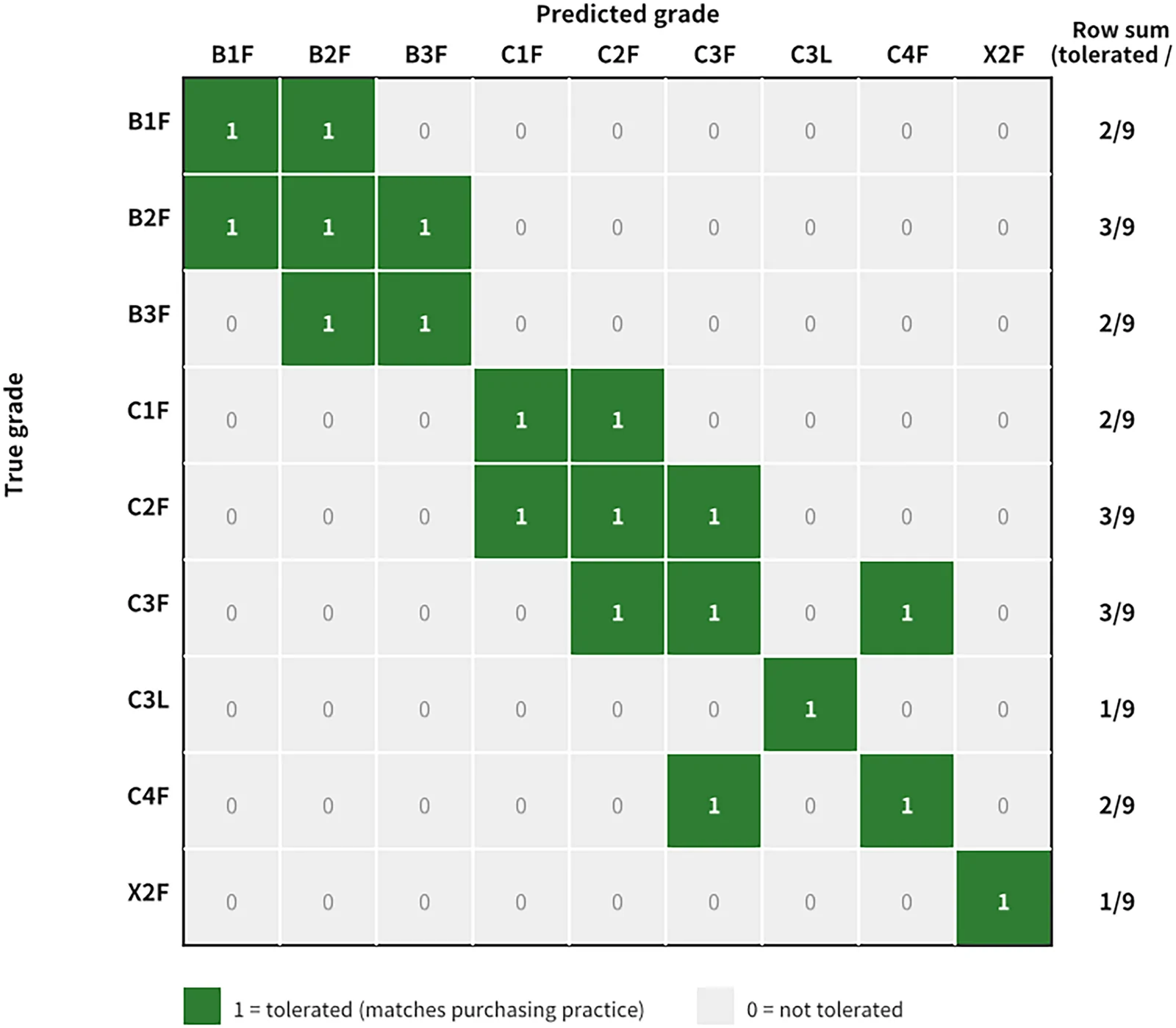
Adjacent-tolerance matrix over the nine main-experiment grades.

The ordinal encoding (position base + quality number) follows the stalk-position structure of GB 2635–1992 (Standardization Administration of China, 1992): leaves are physiologically grouped into lower (X), middle (C) and upper (B) positions, and within each group grades 1–4 form a quality rank; the ordering is therefore a codified domain prior rather than an arbitrary modelling choice. We emphasize, however, that B, C and X primarily denote stalk positions rather than points on a single monotonic quality axis, and that color subclasses such as C3L lie outside any total ordering; the ordinal metrics (QWK, ordinal MAE, CORN) are therefore used only as supplementary descriptors, and the deployment-relevant error measure is the within-group, same-color adjacent-tolerance accuracy defined above. The adjacent-tolerance metric likewise involves no cross-group assumption: it counts only within-group confusions that share the true grade’s position letter and color suffix, so the imposed cross-group ordering cannot inflate it. The supplementary ordinal-loss comparison (**Supplementary Appendix C**, **Supplementary Table C1**) confirms that this choice does not drive our conclusions: replacing cross-entropy with a CORN ordinal loss leaves ordinal MAE essentially unchanged (0.395 vs. 0.407 on the fused model; 0.412 vs. 0.414 on the image-only model) while reducing top-1 accuracy by 2.5–3.9 points, and all primary conclusions of this study are drawn from categorical metrics (top-1 accuracy, balanced accuracy, and macro-F1) that do not involve the ordinal encoding.

### 3.5 Statistical analysis

Seeds measure training stochasticity, not data independence: all seeds of a fold share the same test dates. We therefore (1) average seeds within each fold and report fold means with across-fold standard deviations, and (2) treat the 34 acquisition dates as the independent units in a cluster bootstrap: dates are resampled with replacement 10,000 times, predictions of all seeds on the resampled dates are pooled, and 95% percentile confidence intervals are computed for paired metric differences. We explicitly do not claim “independent runs” from fold × seed combinations.

### 3.6 Annotation and measurement transparency

Because the dataset was acquired from a data vendor, several metadata fields requested by reviewers cannot be verified, and we state them openly rather than omit them: the number, experience, and assignment protocol of the raters; whether raters saw leaf weight when assigning grades; any review or arbitration procedure; inter-rater agreement; camera and scale models, resolution, and calibration schedule; and the timestamp tolerance used in pairing. Two consequences are acknowledged. First, without a human consistency baseline, the 30–36% machine accuracies reported here cannot be mapped onto an irreducible label-noise ceiling; the structured confusion we observe (Section 4.5) is consistent with subjective grade boundaries but does not prove them. Second, if raters could see weight during grading, the weight modality may partially encode human grading priors, which would inflate weight-only accuracy relative to a purely physical interpretation; our reading of weight as a “business prior” rather than a pure physical signal is robust to this caveat, but we flag it explicitly. These items are listed as limitations, and we call for public tobacco-grading benchmarks with documented annotation protocols.

## 4 Results

### 4.1 Effect of split design on apparent grading accuracy

As a preliminary check on the full image pool (26,080 images, 11 grades), an identical fully fine-tuned ResNet-50 trained with the same recipe reaches **86.2%** top-1 under a random image-level split but **43.4%** when whole acquisition dates are held out — a 42.8-point gap attributable solely to the partition (**Figure 6**). This single-split comparison uses different preprocessing than the main experiment and is reported as motivation; the main results below use the strict protocol throughout. We use “batch confounding” rather than “leakage” for this phenomenon: random splits do not prove the model memorizes sessions, but they confound grade recognition with session recognition.

**Figure 6:**
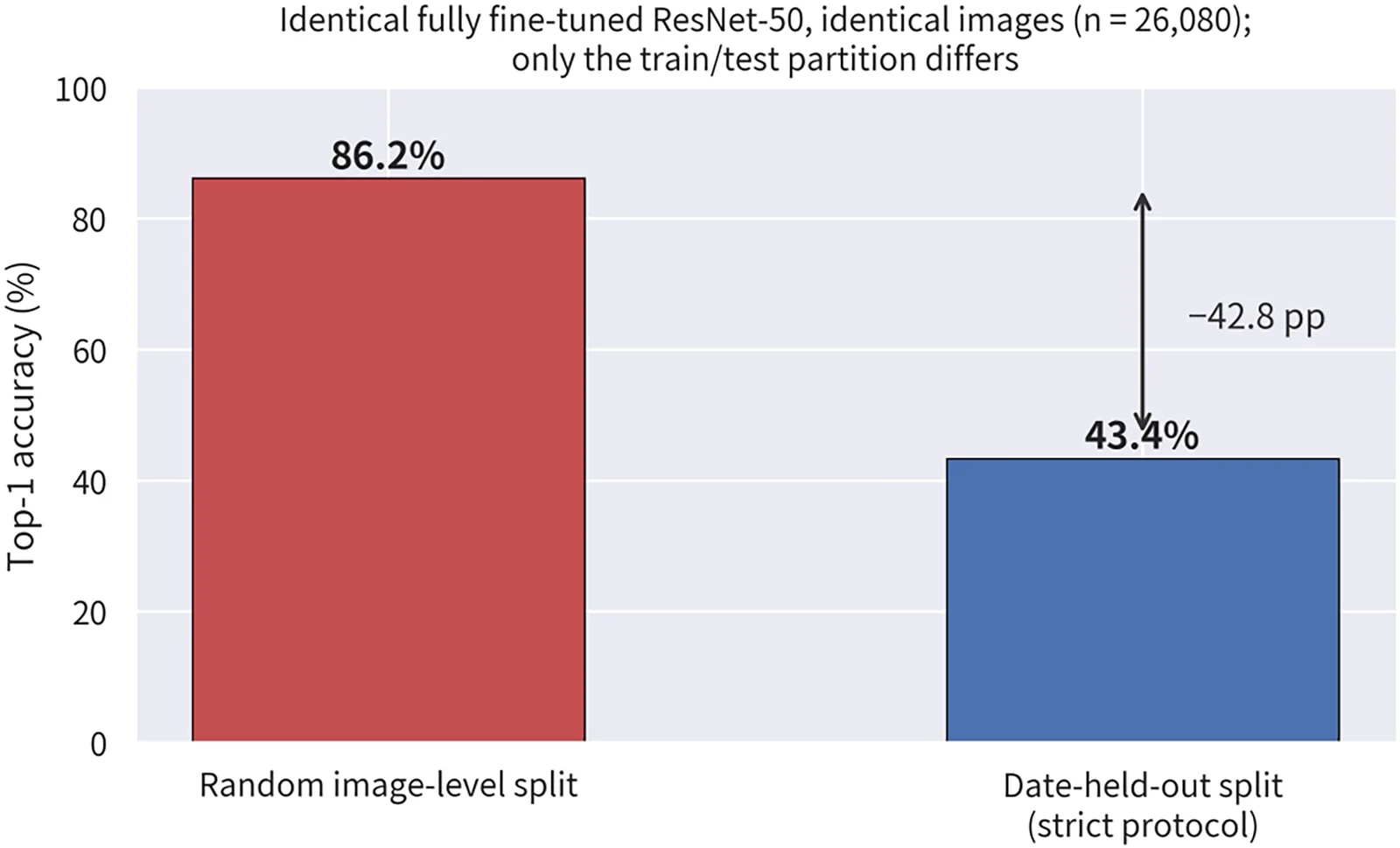
Random vs. date-held-out performance of an identical fully fine-tuned ResNet-50 on identical images (n = 26,080); the 42.8-point gap is attributable to the partition alone.

### 4.2 Nine-grade model comparison under the strict date-held-out protocol

We first evaluate whether incorporating in-line weight records improves tobacco grading performance when all models are tested under the same strict date-held-out protocol. This comparison includes the weight-only model, the vision-only baseline, and four multimodal strategies (hierarchical classification, concatenation fusion, late probability averaging, and gated fusion). All models use identical folds, preprocessing, and training repetitions, allowing the contribution of each modality and fusion mechanism to be assessed without differences in evaluation design. The resulting cross-date performance across multiple classification and ordinal metrics is summarized in **Table 2**.

**Table 2:** Nine-grade cross-date performance (3-fold mean ± std of fold means; seeds averaged within fold).

Model	Top-1	Bal. acc	Macro-F1	QWK	Ord. MAE	Adj. tol
weight	0.248 ± 0.091	0.221 ± 0.069	0.142 ± 0.066	0.074 ± 0.040	0.510 ± 0.134	0.451 ± 0.101
vision	0.346 ± 0.041	0.366 ± 0.028	0.351 ± 0.036	0.514 ± 0.012	0.414 ± 0.033	0.556 ± 0.038
hier	0.348 ± 0.053	0.360 ± 0.039	0.349 ± 0.047	**0.519 ± 0.065**	**0.391 ± 0.020**	0.564 ± 0.049
concat	**0.363 ± 0.044**	**0.391 ± 0.032**	**0.371 ± 0.034**	0.510 ± 0.013	0.407 ± 0.035	0.569 ± 0.030
late	0.361 ± 0.046	0.374 ± 0.030	0.363 ± 0.038	0.512 ± 0.007	0.403 ± 0.044	**0.571 ± 0.047**
gated	0.359 ± 0.031	0.385 ± 0.027	0.363 ± 0.022	0.507 ± 0.026	0.416 ± 0.039	0.564 ± 0.031

Best per column in bold, second best underlined.

From **Table 2**, we have three observations. (1) Fusion of the two modalities occupies the top of every metric, but by a margin of only 1–2 top-1 points over vision alone. (2) Gated fusion is not more accurate than concat or late fusion, but it has the smallest across-fold standard deviation (0.031 vs. 0.044/0.046) — its value is stability, not accuracy. (3) Weight alone is weak on average but extremely variable (± 0.091; fold-wise 34.7%/22.7%/17.0%), foreshadowing the batch-dependent reliability analyzed in Section 4.5. The hierarchical model deserves separate comment: it matches vision on top-1 (0.348 vs. 0.346) and yields the mildest ordinal-error profile of all models (highest QWK 0.519, lowest ord. MAE 0.391), a useful property for pricing-adjacent decision support; but its across-fold variance is the largest among the fused models (± 0.053), consistent with first-stage position-group errors propagating to the second stage on folds where the B/C boundary shifts.

### 4.3 Standalone predictive value of leaf weight across classifiers

To determine whether the predictive information contained in leaf weight depends on the choice of neural architecture, we further compare the weight-based neural model with several classical classifiers using the same date-held-out folds. These baselines provide a direct assessment of the intrinsic grading information carried by the scalar weight signal and help distinguish modality limitations from model-specific effects. The performance of the classical weight-only approaches is reported in **Table 3**.

**Table 3:** Weight-only baselines on identical folds (3-fold mean ± std top-1).

Baseline	Top-1	Macro-F1	Adj. tol
logistic regression	0.259 ± 0.074	0.160	0.451
RBF-SVM	0.252 ± 0.078	0.158	0.451
Gaussian naive Bayes	0.254 ± 0.069	0.154	0.444
gradient boosting	0.238 ± 0.024	0.200	0.409
1-D threshold	0.235 ± 0.031	0.218	0.369
weight MLP (**Table 2**)	0.248 ± 0.091	0.142	0.451

No weight-only classifier exceeds 26% on average; the scalar carries real but limited grade information, and the MLP adds nothing over logistic regression. Weight’s value emerges only in combination with images (Section 4.4).

### 4.4 Statistical robustness of fusion gains across acquisition dates

Although multimodal models achieve slightly higher average accuracy than the vision-only baseline, the magnitude and reliability of these improvements require statistical evaluation under the date-grouped protocol. Because acquisition dates—not individual leaves—are the independent unit of deployment, we run a paired cluster bootstrap at the date level rather than the leaf level. This yields confidence intervals for the performance gap between models without inflating significance by treating correlated same-date samples as independent. The paired comparisons and 95% confidence intervals are presented in **Table 4**.

**Table 4:** Paired differences with 95% percentile CIs (10,000 date-level resamples; seeds pooled within dates).

Pair	Δ Top-1 [95% Ci]	Δ Adj. tol [95% CI]	Significant?
late − vision	+0.015 [+0.002, +0.031]	+0.015 [+0.002, +0.029]	yes
concat − vision	+0.017 [−0.001, +0.032]	+0.013 [−0.004, +0.031]	borderline
gated − vision	+0.013 [−0.002, +0.027]	+0.009 [−0.009, +0.027]	no
gated − concat	−0.004 [−0.018, +0.010]	−0.005 [−0.013, +0.007]	no
late − weight	+0.113 [+0.020, +0.218]	+0.120 [−0.009, +0.264]	yes (top-1)
concat − weight	+0.115 [+0.013, +0.223]	+0.118 [−0.023, +0.272]	yes (top-1)
vision − weight	+0.098 [−0.008, +0.214]	+0.105 [−0.034, +0.259]	no

Fusion beats weight-only decisively (≈ +11 points, CI excludes zero); fusion beats vision by a small margin that is statistically robust only for late fusion. The very wide intervals for weight-involving pairs reflect weight’s cross-date variance, not sample size.

### 4.5 Batch-dependent modality reliability and structured grading errors

Aggregating by test date (102 fold × seed × date cells) reveals that the two modalities’ reliabilities are batch-dependent and complementary. On 2025-09-06 (C3F/X2F/C3L, n = 79), the weight model reached 58.2–60.8% while the image model collapsed to 2.5–3.8%; on 2025-09-01 (five grades, n = 1006), images reached 73.8–75.0% while weight managed only 29.6–30.3%. Extreme single-grade dates (e.g., 2025-09-04, B1F only) trivially produce 95–99% image accuracy and ~0% weight accuracy, and are reported with their grade composition to avoid misreading. Across all 34 dates, per-date image and weight accuracies are weakly *negatively* correlated (Pearson r = −0.179): the modalities do not merely vary in reliability — they tend to fail on different batches (**Figure 7**). On dates with ≥3 grades, gated fusion exceeds vision by +1.6 points on average and in 64% of date cells. The fused model usually stays between the two modalities, but it can collapse below both of them: on 2025-09–06 the gate kept 78–80% of its weight on the failed image stream and gated accuracy (1.3%) fell below even the collapsed image-only model (2.5–3.8%), far below weight-only (≈60%). Consistently, the gate value correlates only weakly with date-level image error (Pearson r = 0.16, Spearman ρ = 0.22; norm-weighted contribution share ≈ 0.81–0.90 for vision throughout). A scalar gate trained without reliability supervision does not learn to detect visual failure — an explicit boundary result against over-claiming adaptive gating.

**Figure 7:**
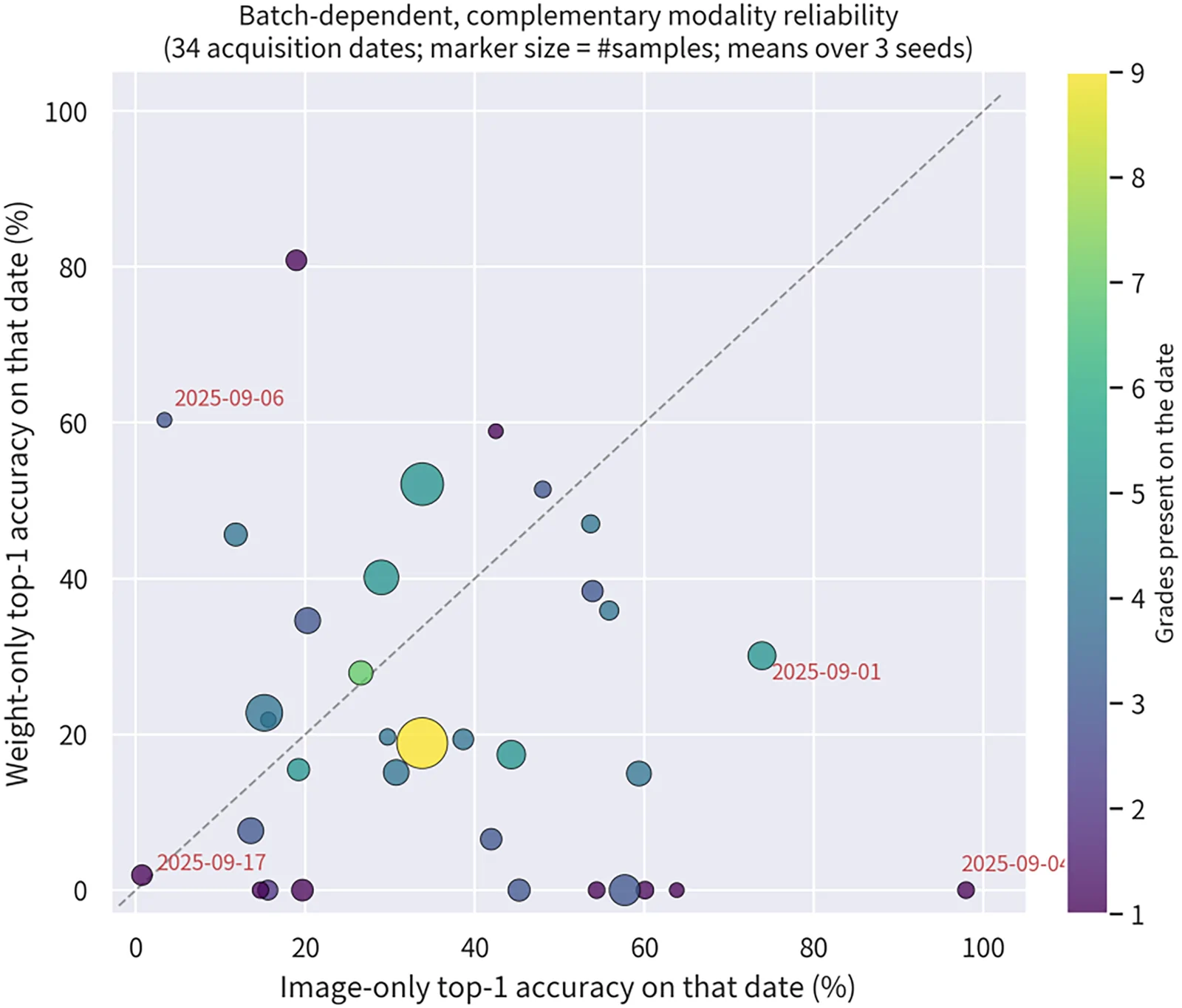
Per-date modality reliability: image-only vs. weight-only top-1 accuracy for each of the 34 acquisition dates (means over 3 seeds; marker size = sample count; color = number of grades present). Points far from the diagonal mark complementary failures.

The out-of-fold confusion matrix (**Figure 8**) confirms that errors are structured rather than random. Per-grade recall ranges from 0.57 (B1F) and 0.53 (B2F) down to 0.24 (C4F) and 0.25 (C3L); confusions concentrate within the same position group between adjacent quality numbers (e.g., C3F → C2F 0.24), and the only notable cross-group confusions sit at group boundaries (C1F → B1F 0.34; C4F → B3F 0.26; X2F → C3F 0.37). This structure directly motivates the hierarchical and ordinal modeling directions discussed in Section 5.

**Figure 8:**
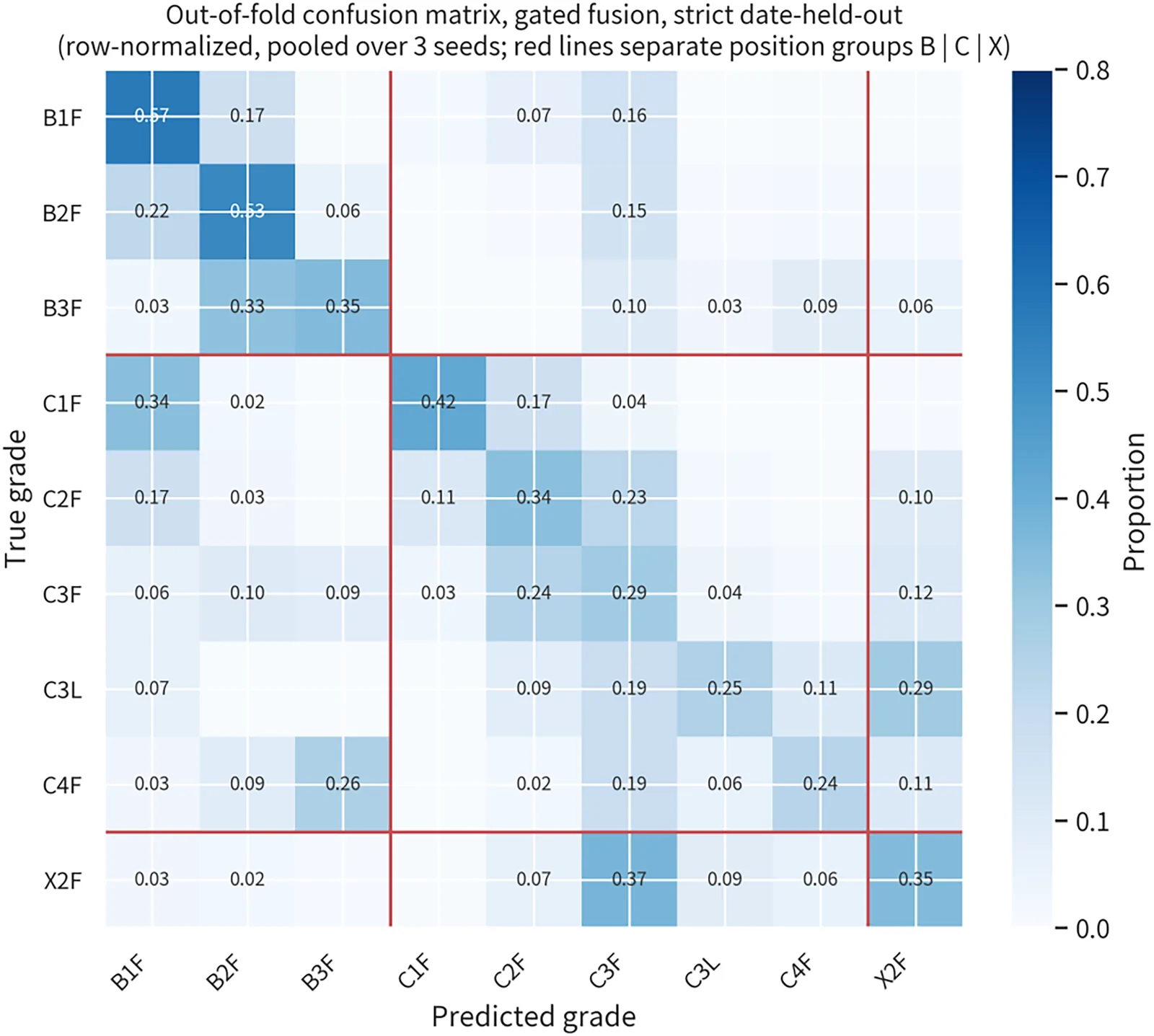
Out-of-fold confusion matrix of gated fusion under the strict protocol (row-normalized, pooled over 3 seeds; red lines separate position groups B | C | X).

Qualitative failure analysis. **Figure 9** compares samples from 2025-09-06 — where the vision model collapsed to 2.5–3.8% while the weight model held at 58–62% — with same-grade samples from dates on which vision works. Grad-CAM maps show that on well-classified dates attention concentrates on the leaf lamina, whereas on 2025-09–06 it drifts to leaf tips, stems and belt markers — non-discriminative, batch-correlated regions — visually confirming the shortcut mechanism. The gate’s behavior on that date is also explained: the gate infers modality reliability from training-period statistics and receives no signal that the image stream will fail on an unseen date, so it retains its ~80% default on the image stream. Reliability-supervised gating, listed as future work, targets exactly this failure mode.

**Figure 9:**
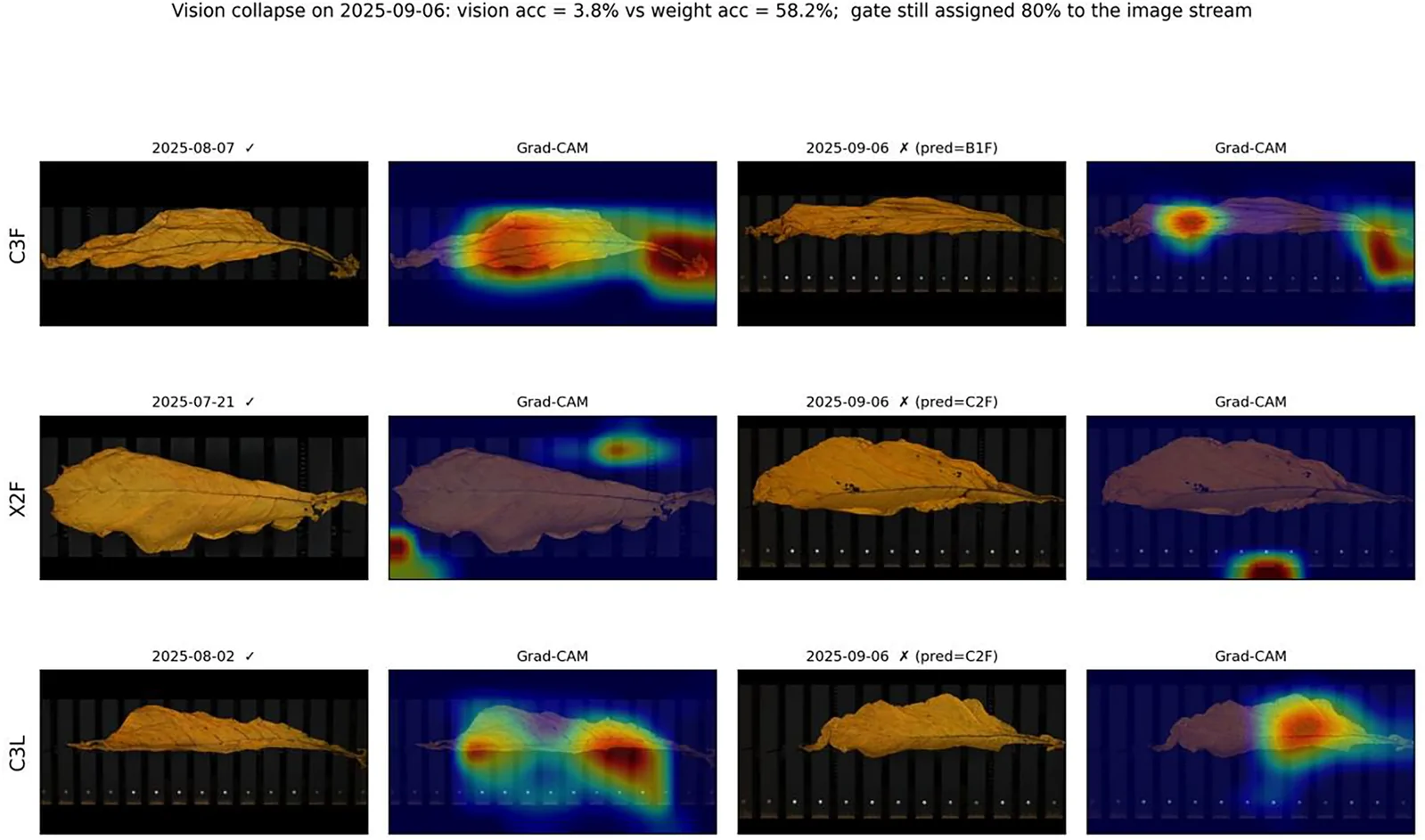
Qualitative failure analysis for 2025-09-06. Rows: grades C3F, X2F, C3L. Per row: a correctly classified same-grade sample from a reference date (left pair) and a misclassified 2025-09–06 sample (right pair), each with its Grad-CAM map. On well-classified dates attention concentrates on the leaf lamina; on 2025-09–06 it drifts to leaf tips, stems and belt markers.

### 4.6 Test-time robustness of gated fusion to noisy and missing modalities

To assess the robustness of multimodal fusion under imperfect sensor inputs, we next conduct test-time perturbation experiments on the gated-fusion model. This model is particularly informative for such an analysis because its learned scalar gate controls the relative contribution of the image and weight representations for each sample. We evaluate three practically relevant input degradations: perturbations of 10% and 20% in the weight measurement, a missing weight record replaced by the training-fold mean, and loss of visual information through image masking. The resulting changes in top-1 accuracy, balanced accuracy, and adjacent-tolerance accuracy are summarized in **Table 5**.

**Table 5:** Test-time ablations on gated fusion (9 runs, mean ± std).

Condition	Top-1	Bal. acc	Adj. tol
clean	0.359 ± 0.030	0.385	0.564
weight + 10% noise	0.358 ± 0.030	0.384	0.563
weight + 20% noise	0.357 ± 0.029	0.383	0.562
weight missing (train mean)	0.304 ± 0.022	0.316	0.536
image masked	0.190 ± 0.032	0.226	0.329

The fused model is robust to realistic scale noise (≤1 point loss at ±20%). When weight is entirely removed, accuracy degrades gracefully to 30.4%, still above weight-only; this indicates partial reliance on image–weight correlations learned during training. Masking the image instead costs 16.9 points, and the residual accuracy (19.0%) falls slightly below weight-only (24.8%) — consistent with Section 4.5, where the gate fails to redirect trust toward weight even when vision carries no signal.

### 4.7 Comparison of backbone fine-tuning regimes under the strict date-held-out protocol

To determine how backbone adaptation depth affects cross-batch grading and to replace our earlier single-split probe comparison (which used a different partition), we re-ran the full pipeline with the backbone fully frozen (linear probe) and fully fine-tuned, using identical folds and seeds (**Table 6**).

**Table 6:** Top-1 accuracy by fine-tuning regime (3-fold mean ± std of fold means; identical splits throughout).

Regime	Vision	Concat	Gated	Late
frozen (linear probe)	0.288 ± 0.046	0.342 ± 0.039	0.334 ± 0.038	0.307 ± 0.045
**partial (layer4; main protocol)**	**0.346 ± 0.041**	**0.363 ± 0.044**	**0.359 ± 0.031**	**0.361 ± 0.046**
full fine-tuning	0.330 ± 0.056	0.343 ± 0.047	0.338 ± 0.044	0.339 ± 0.057

Bold values indicate the best value in each column.

Partial fine-tuning is best for every model. Fully fine-tuning has the highest variance and collapses on the hardest fold (vision fold-0: 0.266 vs. 0.302 for partial), indicating that deep adaptation re-fits training-session appearance that does not transfer across dates; the frozen backbone underfits (vision 0.288). This also explains why our motivating experiment (Section 4.1, full fine-tuning) showed an especially large random-split inflation, and it justifies the partial regime used throughout this paper. We note the ranking is consistent for unimodal and fused models alike, so adaptation depth does not confound the fusion conclusions of Section 4.4.

### 4.8 Supplementary 11-grade evaluation with flagged fallback splits for X3F and X4F

For completeness we re-ran weight, vision, gated, and late fusion on all 11 grades, keeping the strict date-held-out protocol for the nine main grades and using the explicitly flagged fallback splits for the two rare ones: X3F (single date; stratified random fallback) and X4F (two dates; fixed cross-date split, one date fully held out). Overall, 11-grade performance (**Table 7**) is comparable to the main experiment.

**Table 7:** Supplementary 11-grade results (3-fold mean ± std).

Model	Top-1 (11 grades)	Strict-9 subset	X3F recall †	X4F recall ‡
weight	0.229 ± 0.084	—	—	—
vision	0.328 ± 0.023	0.317 ± 0.019	0.92	0.44
gated	0.339 ± 0.029	0.344 ± 0.025	0.90	0.21
late	0.343 ± 0.032	0.337 ± 0.024	0.92	0.38

^†^X3F: single-date stratified random fallback — recall is optimistic and flagged as such. ^‡^X4F: genuine two-date held-out split. Rare-grade recalls are reported separately because their split types differ.

The supplementary results validate, in miniature, the paper’s central warning. The only rare grade that *can* be tested across dates, X4F, reaches just 0.21–0.44 recall, whereas X3F’s 0.90–0.92 recall is an artifact of its single-date random fallback; including such grades in an aggregate metric would silently inflate it, which is precisely why the main experiment (Sections 4.2–4.7) excludes them. Fusion rankings within the 11-grade task mirror the main experiment (late ≈ gated > vision > weight), indicating that the conclusions are not sensitive to the grade inventory.

### 4.9 Cross-backbone validation of split-induced performance inflation

To test whether the collapse documented in Section 4.1 is specific to the ResNet-50 backbone, we re-ran the complete strict experiment with DenseNet-121, the backbone family used in the large-scale grading study of Xin et al. (2023). Every other aspect of the protocol was held identical: the same date-grouped folds, the same three seeds, the same input resolution and augmentation, and partial fine-tuning restricted to the last dense block (mirroring the layer-4-only tuning of Section 3.3). Under the strict protocol, DenseNet-121 reaches 0.338 (vision), 0.343 (concat), 0.354 (gated), and 0.348 (late fusion) top-1 accuracy (**Table 8**). Every fusion variant falls within one cross-fold standard deviation of the corresponding ResNet-50 figure, with the same fusion ordering (gated ≈ late > concat > vision) and the same hardest fold (fold 0). Under stratified random splitting of the very same 24,272 samples, the identical DenseNet-121 vision model reaches 0.715 top-1 (a 37.6-point inflation); the ResNet-50 vision model, re-measured under the same random-split protocol, reaches 0.761 ± 0.007 (a 41.5-point inflation), closely matching the 42.8-point gap of the preliminary full fine-tuning experiment in Section 4.1. The performance collapse under batch-respecting evaluation is therefore architecture-independent: it is a property of the evaluation protocol, not of the backbone.

**Table 8:** Cross-backbone comparison.

Backbone	Vision	Concat	Gated	Late	Vision (Random)	Gap (Points)
ResNet-50	0.346 ± 0.041	0.363 ± 0.044	0.359 ± 0.031	0.361 ± 0.046	0.761 ± 0.007	41.5
DenseNet-121	0.338 ± 0.036	0.343 ± 0.044	0.354 ± 0.035	0.348 ± 0.034	0.715 ± 0.011	37.6

Strict-protocol columns report top-1 accuracy (mean ± standard deviation across the three date-held-out folds); the random column reports top-1 under stratified random splitting; the gap is the random-split accuracy minus the strict-protocol accuracy, in percentage points. Both rows are measured entirely on the nine-grade pool of 24,272 samples; the ResNet-50 random-split arm uses the same protocol as the DenseNet-121 random arm (three seeds, stratified 70/15/15 splits, partial fine-tuning).

Both random-split arms use stratified 70/15/15 random splits of the same 24,272 samples with partial fine-tuning (ResNet-50: layer 4; DenseNet-121: last dense block); mean ± standard deviation over the three seeds. “late” denotes 0.5·vision + 0.5·weight probability averaging, as in **Table 2**.

### 4.10 Disentangling split-induced inflation from domain shift on a shared external test set

The headline gap between random-split (86.2%) and date-held-out (43.4%) accuracy conflates two factors: information usable through date overlap, and the intrinsic difficulty of unseen-date domain shift. To decompose them, we held out 10 acquisition dates as an external test set invisible to all training, and trained on the remaining 24 dates under both protocols (three repetitions): model A with a random 80/10/10 split, and model B with a date-held-out 18/6 split, plus model C, a random-split model whose training set was subsampled to exactly match B’s size (controlling the training-set-size confound of the 12/11/11 rotation). Both models were evaluated on the same external dates (**Table 9**). Model A reaches 75.0% on its internal random test set but only 37.9% on the external dates — a ~37-point drop within a single trained model, isolating the contribution of date overlap. On the shared external dates, A (37.9%) and B (31.4%) are statistically comparable, so the high internal figures are inflation rather than evidence of an easier task. Model C (74.4% internal) matches A, showing that the reduced training set of the strict protocol does not explain the collapse. A linear probe further predicts the acquisition date from the learned image features with 91.9–97.3% accuracy (chance ≈ 9%), while the same features predict the grade on unseen dates at only 26.6–33.9%: the representation encodes batch identity more strongly than grade identity, providing direct mechanistic evidence of batch confounding.

**Table 9:** Decomposition of split-induced inflation on shared external test dates (mean ± std over 3 repetitions; top-1/balanced accuracy, %).

Training model	Evaluation set	Train size	Top-1 (%)	Balanced acc (%)
Random-split training (A)	internal random test	13736	75.0 ± 4.6	75.2 ± 5.4
Random-split training (A)	external dates (shared)	13736	37.9 ± 5.5	43.0 ± 5.3
Random-split, size-matched (C)	internal random test	11389	74.4 ± 3.3	74.4 ± 4.1
Random-split, size-matched (C)	external dates (shared)	11389	38.6 ± 4.3	42.6 ± 4.9
Date-held-out training (B)	held-out val dates	11389	37.4 ± 10.9	37.2 ± 7.0
Date-held-out training (B)	external dates (shared)	11389	31.4 ± 6.5	34.9 ± 2.4

### 4.11 Station- and cultivar-controlled analysis

Because acquisition date co-varies with station, cultivar and environmental conditions, we repeated the evaluation restricted to test samples whose station and cultivar both appeared in the training dates. Accuracy in this covariate-controlled subset remains 29.7–35.1% (sample-level; fold range), far below the random-split figure, indicating that the collapse occurs within the same station and cultivar and is driven by temporal/batch factors rather than by station or cultivar substitution. We therefore frame the protocol as controlling the production batch — the joint bundle of covariates carried by a date — which is the granularity at which deployment generalization must hold.

### 4.12 Estimated information limits of the in-line weight modality

To quantify how much a single scalar can contribute, we computed empirical estimates of the weight modality’s attainable contribution (**Table 10**). A Bayes-optimal classifier with per-grade Gaussian weight distributions reaches 20.9–33.4% with training-estimated parameters, and only 27.4–37.4% even when the Gaussians are fitted on the test dates themselves (an unattainable oracle). The histogram-estimated mutual information I(W;G) ≈ 0.45 bit covers only ~14% of the 3.17 bits required to identify a grade. Even a per-sample oracle that ‘knows’ which modality is correct on every test sample reaches only 49.3% (vision 32.8%), and a per-(date × grade) oracle 46.2%. The small fusion gains reported in Section 4.2 therefore reflect the modality’s limited information content as estimated here, rather than the choice of fusion mechanism; we stress that these are estimator-dependent estimates under the stated Gaussian and histogram assumptions, not general theoretical upper bounds on multimodal fusion; realizing even part of the oracle gap would require per-sample reliability knowledge that is not observable at inference time, motivating reliability-supervised fusion as future work.

**Table 10:** Estimated information limits of the in-line weight modality (nine grades; chance ≈ 11.1%).

Estimator	Top-1/value	Note
Bayes-optimal weight classifier (train-fitted Gaussians)	20.9–33.4%	fold range on strict folds
Bayes-optimal weight classifier (test-fitted oracle)	27.4–37.4%	unattainable upper bound
Mutual information I(W;G)	≈ 0.45 bit	vs 3.17 bit needed to identify the grade
Per-(date × grade) oracle fusion	46.2%	knows which modality is better per group
Per-sample oracle fusion	49.3%	knows per-sample correctness (vision alone: 32.8%)

### 4.13 Stronger fusion and alignment baselines

To test whether deeper fusion or explicit domain alignment changes the picture, we added three baselines under the identical strict protocol (three seeds): FiLM feature-level conditioning of the image features by the weight embedding, cross-modal attention pooling of the spatial feature map with the weight embedding as query, and date-adversarial training with a gradient-reversal date classifier (**Table 11**). FiLM (36.6%) and cross-modal attention (36.7%) improve significantly over the vision baseline (paired t-tests over the 102 fold × seed × date cells, p < 0.005). Note that **Table 11** aggregates nine independent seed × fold runs, whereas **Table 2** aggregates fold means, so the baseline values differ slightly between the two tables; all comparisons here use the common run-level pool. Relative to late fusion (33.7%), the run-level gains are +2.9 and +3.0 points, but the paired per-date-cell differences (per-date breakdown in **Supplementary Table C2**, **Supplementary Appendix C**) are smaller (+1.0 and +0.9 points) and not significant (p = 0.23 and p = 0.25), confirming that the fusion-strategy space is saturated near the modality’s estimated information limit (Section 4.12). Date-adversarial training reduces accuracy to 29.3% — significantly worse than late fusion (−4.3 points, p = 0.028) — showing that forcing date invariance destroys grade semantics (negative transfer), consistent with the entangled grade–batch composition documented in **Supplementary Appendix A**.

**Table 11:** Stronger fusion and alignment baselines under the strict date-held-out protocol (3 seeds × 3 folds, mean ± std, %).

Model	Type	Top-1 (%)	Balanced acc (%)
vision	image only	32.3 ± 4.3	35.3 ± 4.6
weight	weight only	24.3 ± 7.4	21.0 ± 5.8
concat	back-end feature fusion	34.9 ± 3.9	37.8 ± 2.2
gated	back-end scalar gating	34.2 ± 3.3	38.1 ± 2.7
late	back-end probability averaging	33.7 ± 4.4	35.9 ± 4.5
film	feature-level FiLM conditioning (new)	36.6 ± 4.3	39.3 ± 2.5
xattn	cross-modal attention pooling (new)	36.7 ± 3.7	39.1 ± 2.4
adv	date-adversarial alignment (new)	29.3 ± 7.1	30.7 ± 8.7

## 5 Discussion

### 5.1 Interpretation of performance under strict cross-date evaluation

Taken together, the experiments in Sections 4.1–4.13 show that the apparent difficulty of tobacco grading depends strongly on the evaluation protocol. Under an evaluation that respects the batch structure of purchasing-line data, fine 9-grade tobacco grading is a 35% top-1 problem, not an 86–99% one. This is not a failure of our models; it is the honest difficulty of the task when session appearance cannot be exploited. Errors concentrate within position groups and between adjacent quality numbers, showing high adjacent-tolerance (55–57%) and moderate QWK (≈ 0.51). This structured confusion indicates genuine grade-relevant learning and aligns with the known subjectivity of adjacent-grade boundaries in GB 2635 practice. We emphasize that lower absolute accuracy under our strict evaluation should not be interpreted as evidence that our models are weaker. Instead, we believe that previously reported high (80–99%) results may be influenced by evaluation protocols that allow overlap between training and test batches, leading to an optimistic estimate of generalization across production batches. We tested this explanation directly. Re-running DenseNet-121 [the backbone reported in Xin et al. (2023)] under our date-grouped protocol with identical folds, seeds, and training recipe (Section 4.9, **Table 8**) yields 33.8–35.4% top-1 across fusion variants, which is statistically indistinguishable from ResNet-50. In contrast, the same vision model reaches 71.5% under random splitting of the identical samples, a 37.6-point inflation, matching the evaluation-dependent gap we observe for ResNet-50 (Section 4.1). This performance collapse reflects the evaluation protocol, not the backbone choice. Because samples within the same acquisition date share similar lighting, moisture, and cultivar conditions, random splitting may allow this batch-level signal to be partially reused and inflate measured accuracy for any model. The decomposition experiment of Section 4.10 attributes the random-versus-strict gap quantitatively: about 37 points arise within a single random-split model when date overlap is removed, and models trained under either protocol converge to 31–38% on the same unseen dates. Previously reported 80–99% figures should therefore be read as batch-confounded estimates unless date-level overlap is excluded.

### 5.2 Contribution and limitations of the weight modality

Weight adds a statistically robust 1.5 points through simple probability averaging, survives ±20% sensor noise, and occasionally rescues entire batches when vision fails. But its reliability is itself batch-dependent (fold range 17–35%), plausibly because origin, cultivar, moisture, and scale state shift the weight–grade mapping across dates. If raters could see weight while labeling (Section 3.6), part of its predictive power may also reflect human priors rather than physics. We therefore describe weight as a business prior with real complementary value — not a silver bullet. We also emphasize that statistical significance and practical significance are distinct here: whether a 1.5-point gain justifies deploying and maintaining a fusion pipeline depends on the economic value of correct grading decisions at a given purchasing station. Because our approach reuses weight sensors already installed on the conveyor line, its marginal hardware cost is zero; the added cost is limited to model maintenance and the small additional inference latency of a second branch. In high-throughput stations that process thousands of leaves per shift, even a small reduction in misgrading (about 1–2 percentage points) could reduce downstream price-adjustment disputes. However, we do not have station-level cost data to quantify this impact directly. Instead, we report the effect size and its confidence interval, so each station can judge its practical value using its own cost structure.

### 5.3 Fusion strategy and the limits of scalar gating

Late fusion, which requires no joint training, is the only strategy whose gain over vision excludes zero in the date-level bootstrap. Gating does not beat concatenation in accuracy but halves cross-fold variance, which has operational value when deployment conditions drift. The negative result that a scalar gate does not detect visual failure (r = 0.16) implies that reliability-aware fusion needs explicit supervision or uncertainty signals. To explore this further, we ran a supplementary experiment in **Supplementary Appendix B**. We added the image model’s predictive entropy to the gate input as an uncertainty signal, instead of using only the learned embedding. The result was negative in an instructive way: the gate–error correlation did not improve consistently (per-fold means −0.38, −0.30, and +0.62, the sign itself flipping across folds; nine-run mean ≈ 0), and accuracy was unchanged relative to plain gating (36.4% vs. 35.9% top-1). We tested whether explicit uncertainty features could enable reliability-based weighting. The gate did not learn it. This confirms that fusion models need explicit supervision (e.g., error-prediction targets), not just better inputs. Relatedly, because GB 2635 grades are ordinal and a substantial share of our models’ errors fall on an adjacent grade, we also tested replacing standard categorical cross-entropy with an ordinal-aware loss (CORN; **Supplementary Appendix C**). The ordinal objective reduced ordinal MAE only marginally on the fused model (0.395 vs. 0.407) and had little effect on the image-only model (0.412 vs. 0.414). However, it cost 2.5–3.9 points of top-1 accuracy, which is an unfavorable trade-off under batch shift. We therefore retain standard cross-entropy and report ordinal behavior using adjacent-tolerance and QWK. Whether stronger ordinal formulations (e.g., label smoothing toward adjacent grades or ordinal heads trained with reliability supervision) can avoid this trade-off remains an open question.

### 5.4 Methodological and practical implications

Our findings support three reporting practices for agricultural grading studies with batch-structured data: (i) the grouping unit should be explicitly defined and checked for split overlap; (ii)–repeated seeds should be treated as measures of training variability rather than independent observations; (iii) fold assignments should be published to enable verification and reproduction. Deployment claims should also distinguish the absence of additional sensing hardware from zero total deployment cost. Although the existing in-line scale avoids the need for a new sensor and the **Supplementary Appendix D** benchmark indicates only a small inference overhead for the weight branch, integration, calibration, and maintenance remain relevant. Deployment decisions should therefore consider local line speed, workflow, and the practical value of the modest performance gains reported in Sections 4.2 and 4.4.

### 5.5 Limitations and future validation

Right now, we cannot fully tell whether the model’s mistakes come from differences in human grading or from the model itself, because the dataset does not include repeated independent ratings or any measure of agreement between graders. For a more reliable conclusion, future work should re-check part of the test set with multiple certified graders and report an inter-grader agreement score (e.g., kappa). Until then, the proposed method should be used as an aid to support re-inspection, not as a fully independent grading system. Several further limitations apply. The grading workflow of the purchased dataset could not be verified: the number of raters, any re-inspection or adjudication procedure, and whether raters could see scale readings while grading are unknown; if readings were visible, part of the weight signal may encode human priors rather than plant physics. We note, however, that the weight channel does not behave like a label-leakage channel: a weight-only classifier reaches only 24–26% and I(W; G) ≈ 0.45 bit (Section 4.12), far below what encoding of grading decisions would imply. Station metadata are missing for 21 of the 34 dates (**Supplementary Appendix A**), and camera/scale hardware specifications were unavailable from the vendor. Moreover, because station, cultivar and environmental conditions co-vary with acquisition date in this dataset, their individual effects cannot be fully separated from those of date; the controlled analysis of Section 4.11 bounds their contribution within shared station–cultivar strata. Finally, grade coverage is limited: the strict rotation retains the nine grades with at least three acquisition dates, so the X group is represented by X2F alone and the corresponding branch of the hierarchical model is trivial; validation across additional stations, seasons and the full GB 2635 grade set is therefore required before deployment, and is listed as future work (Section 6). Without a human-consistency baseline, the 30–36% machine accuracies cannot be mapped onto an irreducible label-noise ceiling; published inter-rater agreement for manual tobacco grading suggests that human consistency itself is limited, so the machine figures may be closer to the achievable ceiling than the absolute numbers imply.

## 6 Conclusions

We fused existing weight records with conveyor-belt images to enable automated tobacco grading on the purchasing line. Under a strict date-held-out validation protocol, this fusion approach improved classification accuracy from 34.6% (vision alone) to 36.1%–36.3%. Among the four fusion architectures of the main comparison, late fusion was the only statistically robust improvement (+1.5%, 95% CI [+0.2, +3.1]); the deeper FiLM and cross-modal-attention baselines added in Section 4.13 improved over vision alone but not beyond late fusion, and gated fusion showed the most stable performance across batches. However, we found that the two modalities fail in complementary, batch-dependent ways, and a simple gate cannot automatically detect these failures. This points to three clear directions for improvement: more intelligent fusion strategies (reliability-supervised learning), better model architectures (ordinal or hierarchical heads), and cross-season validation. The evaluation protocol and complete date-level fold assignments are reported in the manuscript to facilitate transparent evaluation and fair comparison. The analysis code will be deposited in a public repository upon acceptance to facilitate reproducibility and fair comparison. Looking forward, four directions follow directly from these findings: (i) cross-season validation on the next purchasing season to test temporal stability beyond a single year; (ii) reliability-supervised fusion, in which the gate is trained with explicit per-grade, per-date reliability targets rather than training-period statistics alone; (iii) refined ordinal objectives that respect the position-group hierarchy (X/C/B) and within-group quality ranks; and (iv) construction of a benchmark dataset with complete metadata — station, cultivar, camera and scale specifications, and multi-rater annotations — so that the confounders identified here can be controlled and a human-consistency ceiling established.

## Data Availability

The raw data supporting the conclusions of this article will be made available by the authors, without undue reservation.
