# Supplementary material for: Fusing conveyor-belt images with existing in-line weight records for flue-cured tobacco leaf grading: a strict date-held-out evaluation
Source: Front Plant Sci. 2026 Oct 26;17:1970460. doi: 10.3389/fpls.2026.1970460 (PMC13745679; doi:10.3389/fpls.2026.1970460)
Supplement: Supplementary file 1 [file SupplementaryFile1.docx]

**Supplementary Material**

**Fusing Conveyor-Belt Images with Existing In-Line Weight Records for Flue-Cured Tobacco Leaf Grading: A Strict Date-Held-Out Evaluation**

Qi Xiong, Wuke Li and Wenxue Tan

Frontiers in Plant Science

**Supplementary Appendix: Sections A–E and Supplementary Tables A1–E2**

# 1 Supplementary Appendix A

Complete date-level fold assignment underlying the strict protocol (Supplementary Table A1). Every acquisition date appears in exactly one partition per fold and is tested exactly once across the three folds; zero train/validation/test date overlap is asserted programmatically.

Supplementary Table A2 (provided as Supplementary File S1) lists the complete grade × acquisition-date composition matrix for all 34 dates and 11 grades; the support matrix for the nine main grades is visualised in Figure 1 (main text). Twenty-one dates carry an 'unknown' station label; their grade compositions and weight distributions (Section 3.1) show no anomaly beyond the missing label itself, and all conclusions hold when these dates are analysed separately (Section 4.11).

# 2 Supplementary Appendix B

We test whether providing the gate with explicit uncertainty signals enables reliability-based weighting (Section 5). The entropy-augmented gate (egated) extends the scalar gate of Section 3.3 with the predictive entropy and maximum-probability confidence of both modality streams, computed from auxiliary classification heads trained jointly with the main head (loss = CE_main + 0.5·CE_vision + 0.5·CE_weight; uncertainty features are detached before entering the gate): $g = \sigma\left( w\cdot[v; q; H_{v}, \text{conf}_{v}, H_{w}, \text{conf}_{w}] \right)$. Folds, seeds, and the training recipe are identical to the main experiment. An adaptive gate should down-weight the vision stream on dates where vision fails — i.e., yield a negative correlation between the date-level mean gate value and the date-level vision error rate. As Supplementary Table B1 shows, accuracy matches concat (0.364 ± 0.038 vs. 0.363 ± 0.044) and slightly exceeds plain gating (0.359 ± 0.031), but the gate–error correlation does not stabilize: per-fold means are −0.38 (mildly adaptive), −0.30 (mixed), and +0.62 (strongly anti-adaptive), with a nine-run mean of −0.02. Explicit uncertainty features alone therefore do not yield reliability-aware gating under batch shift; reliability supervision appears necessary.

# 3 Supplementary Appendix C

Because GB 2635 grades form an ordinal scale (position group, then quality number), we evaluated whether an ordinal-aware training objective improves the ordinal error profile relative to standard categorical cross-entropy (CE). We used CORN (conditional ordinal regression for neural networks): the classifier head outputs K − 1 = 8 cumulative binary logits, is trained with the average binary cross-entropy of the tasks “grade > k”, and predicts the number of thresholds passed. Grades are ordered by position group (X < C < B) and quality number, matching the ordinal encoding of Section 3.4. Folds, seeds, backbone, and the training recipe are identical to the main experiment. Supplementary Table C1 shows that CORN reduces ordinal MAE only marginally on the fused model (0.395 vs. 0.407 for CE) and not at all on the image-only model (0.412 vs. 0.414), while top-1 accuracy drops by 2.5 (concat) to 3.9 (vision) points and QWK degrades for both. Under batch shift, the K − 1 independent binary heads plateau earlier than the CE head, which likely explains the unfavorable trade-off; we therefore retain CE training and characterize ordinal behavior through adjacent-tolerance accuracy and QWK.

# 4 Supplementary Appendix D

Inference benchmark of the deployment configuration (ResNet-50 with partial fine-tuning; 23.5M parameters, 15.0M trainable; 89.7 MB in fp32; the concat fusion head adds 0.5M parameters and 2.1 MB). Timings were measured on the target workstation (NVIDIA RTX 3060, 12 GB; PyTorch 1.8) over 100 iterations after 20 warm-up iterations (Supplementary Table D1). Peak GPU memory during batch-32 inference was 859 MB. In batch-1 operation the fused model processes 108–117 leaves per second with the weight branch contributing only 0.2–0.6 ms of additional latency per leaf; even CPU-only single-leaf inference (204 ms per leaf) stays far below the 1–3 s per-leaf cycle time of a manual purchasing line (weighing plus grading), leaving two to three orders of magnitude of headroom on GPU. Throughput is therefore not a binding constraint for decision-support deployment.

# 5 Supplementary Appendix E

Supplementary Table E1 lists the notation used in the Model section; Supplementary Table E2 specifies every learnable block.

**Supplementary Table A1. Per-date roles in the 3-fold rotation (34 acquisition dates)**

| Date | Site(s) | Samples | Grades | Fold 1 | Fold 2 | Fold 3 |
| --- | --- | --- | --- | --- | --- | --- |
| 2025-07-02 | muyang | 456 | 3 | train | val | test |
| 2025-07-03 | muyang | 176 | 1 | train | val | test |
| 2025-07-18 | muyang | 492 | 1 | train | val | test |
| 2025-07-19 | muyang | 419 | 1 | val | test | train |
| 2025-07-21 | muyang | 256 | 4 | val | test | train |
| 2025-07-23 | muyang | 821 | 3 | val | test | train |
| 2025-07-24 | muyang | 368 | 2 | val | test | train |
| 2025-07-30 | muyang | 227 | 1 | val | test | train |
| 2025-07-31 | unknown | 73 | 1 | test | train | val |
| 2025-08-01 | unknown | 678 | 7 | val | test | train |
| 2025-08-02 | dp | 733 | 4 | test | train | val |
| 2025-08-07 | unknown | 314 | 4 | test | train | val |
| 2025-08-08 | unknown | 175 | 3 | test | train | val |
| 2025-08-10 | unknown | 70 | 1 | train | val | test |
| 2025-08-12 | unknown | 96 | 4 | train | val | test |
| 2025-08-18 | unknown | 4017 | 9 | train | val | test |
| 2025-08-19 | dp/unknown | 2741 | 5 | val | test | train |
| 2025-08-22 | dp/unknown | 591 | 4 | train | val | test |
| 2025-08-24 | unknown | 1938 | 4 | test | train | val |
| 2025-08-26 | unknown | 474 | 3 | test | train | val |
| 2025-08-27 | unknown | 528 | 5 | train | val | test |
| 2025-08-28 | unknown | 1709 | 5 | test | train | val |
| 2025-08-29 | dp | 161 | 1 | train | val | test |
| 2025-09-01 | unknown | 1006 | 5 | val | test | train |
| 2025-09-02 | dp/unknown | 1062 | 5 | test | train | val |
| 2025-09-04 | dp | 177 | 1 | val | test | train |
| 2025-09-05 | dp/unknown | 821 | 3 | test | train | val |
| 2025-09-06 | unknown | 79 | 3 | train | val | test |
| 2025-09-07 | unknown/zhulin | 1300 | 3 | train | val | test |
| 2025-09-08 | zhulin | 799 | 4 | val | test | train |
| 2025-09-10 | unknown | 422 | 4 | test | train | val |
| 2025-09-12 | unknown | 522 | 3 | val | test | train |
| 2025-09-17 | unknown | 415 | 1 | test | train | val |
| 2025-09-22 | dp | 156 | 4 | test | train | val |

Supplementary Table B1. Entropy-augmented gating under the strict protocol (3 folds × 3 seeds). r is the Pearson correlation between the date-level mean gate value and the date-level vision error rate (negative = adaptive); ρ is the Spearman correlation. The final row averages seeds within folds and reports the three-fold mean.

| Fold | Seed | Top-1 | QWK | Ord. MAE | Adj. tol | r | ρ |
| --- | --- | --- | --- | --- | --- | --- | --- |
| 0 | 42 | 0.318 | 0.506 | 0.392 | 0.546 | -0.37 | -0.41 |
| 0 | 7 | 0.332 | 0.515 | 0.385 | 0.556 | -0.41 | -0.35 |
| 0 | 2025 | 0.331 | 0.504 | 0.385 | 0.559 | -0.35 | -0.33 |
| 1 | 42 | 0.406 | 0.538 | 0.389 | 0.606 | -0.04 | +0.08 |
| 1 | 7 | 0.396 | 0.524 | 0.395 | 0.601 | -0.62 | -0.12 |
| 1 | 2025 | 0.403 | 0.541 | 0.392 | 0.604 | -0.23 | -0.06 |
| 2 | 42 | 0.375 | 0.485 | 0.465 | 0.542 | +0.57 | +0.44 |
| 2 | 7 | 0.364 | 0.495 | 0.450 | 0.551 | +0.61 | +0.53 |
| 2 | 2025 | 0.348 | 0.505 | 0.455 | 0.529 | +0.69 | +0.81 |
| **Mean** |  | **0.364** | **0.513** | **0.412** | **0.566** | **-0.02** | **+0.06** |

**Supplementary Table C1. CORN ordinal-loss training vs. standard cross-entropy (CE, from Table 2) under the strict protocol (3 folds × 3 seeds; seeds averaged within folds, then three-fold mean).**

| Mode | Fold | Seed | Top-1 | QWK | Ord. MAE | Adj. tol |
| --- | --- | --- | --- | --- | --- | --- |
| vision | 0 | 42 | 0.273 | 0.433 | 0.389 | 0.521 |
| vision | 0 | 7 | 0.269 | 0.428 | 0.405 | 0.511 |
| vision | 0 | 2025 | 0.260 | 0.442 | 0.404 | 0.502 |
| vision | 1 | 42 | 0.340 | 0.492 | 0.360 | 0.584 |
| vision | 1 | 7 | 0.338 | 0.490 | 0.382 | 0.584 |
| vision | 1 | 2025 | 0.331 | 0.468 | 0.389 | 0.567 |
| vision | 2 | 42 | 0.309 | 0.449 | 0.462 | 0.504 |
| vision | 2 | 7 | 0.325 | 0.479 | 0.446 | 0.519 |
| vision | 2 | 2025 | 0.320 | 0.432 | 0.470 | 0.500 |
| concat | 0 | 42 | 0.275 | 0.437 | 0.405 | 0.527 |
| concat | 0 | 7 | 0.290 | 0.461 | 0.396 | 0.533 |
| concat | 0 | 2025 | 0.282 | 0.466 | 0.407 | 0.488 |
| concat | 1 | 42 | 0.374 | 0.508 | 0.350 | 0.619 |
| concat | 1 | 7 | 0.384 | 0.505 | 0.355 | 0.618 |
| concat | 1 | 2025 | 0.386 | 0.510 | 0.352 | 0.615 |
| concat | 2 | 42 | 0.369 | 0.508 | 0.421 | 0.557 |
| concat | 2 | 7 | 0.360 | 0.507 | 0.423 | 0.549 |
| concat | 2 | 2025 | 0.319 | 0.423 | 0.448 | 0.509 |
| **vision mean** |  |  | **0.307** | **0.457** | **0.412** | **0.532** |
| vision CE (ref.) |  |  | 0.346 | 0.514 | 0.414 | 0.556 |
| **concat mean** |  |  | **0.338** | **0.480** | **0.395** | **0.557** |
| concat CE (ref.) |  |  | 0.363 | 0.510 | 0.407 | 0.569 |

Supplementary Table C2. Per-date paired top-1 accuracy differences between each strong baseline and late fusion (percentage points). Each acquisition date appears in the test partition in exactly one fold of the 3-fold rotation; each row averages that date’s 3 paired seed-level cells (34 dates × 3 seeds = 102 cells in total). n is the number of test samples of the date in a single run. The date-equal column means (last row) are the paired per-date-cell differences reported in Section 4.13 (+1.0 and +0.9 points). They differ from the run-level differences in Table 11 (+2.9 and +3.0 points) because the run-level means weight every test sample equally—so that large dates dominate—whereas the paired significance tests weight every test date equally.

| Date | n | FiLM − late | Cross-modal attention − late | Date-adversarial − late |
| --- | --- | --- | --- | --- |
| 2025-07-02 | 456 | −1.5 | −1.5 | −14.5 |
| 2025-07-03 | 176 | +6.4 | −1.5 | −32.0 |
| 2025-07-18 | 492 | 0.0 | +2.9 | −4.1 |
| 2025-07-19 | 419 | −14.8 | −14.3 | −12.0 |
| 2025-07-21 | 256 | 0.0 | +1.8 | −1.4 |
| 2025-07-23 | 821 | +1.5 | −0.3 | −10.8 |
| 2025-07-24 | 368 | +9.8 | +8.7 | +1.0 |
| 2025-07-30 | 227 | +21.9 | +17.0 | +21.1 |
| 2025-07-31 | 73 | −13.2 | −5.9 | −43.8 |
| 2025-08-01 | 53 | −4.5 | −3.9 | −12.4 |
| 2025-08-02 | 733 | −3.4 | +1.9 | −29.0 |
| 2025-08-07 | 314 | −3.1 | −3.5 | −9.7 |
| 2025-08-08 | 175 | −3.2 | −7.6 | −11.6 |
| 2025-08-10 | 70 | 0.0 | −1.4 | −28.6 |
| 2025-08-12 | 96 | −1.0 | −0.7 | +15.3 |
| 2025-08-18 | 4017 | +7.1 | +6.3 | −7.2 |
| 2025-08-19 | 2741 | +7.3 | +6.7 | +1.7 |
| 2025-08-22 | 578 | −10.4 | −10.9 | +8.6 |
| 2025-08-24 | 1938 | −2.6 | −0.7 | −3.1 |
| 2025-08-26 | 474 | −8.3 | +1.7 | +0.1 |
| 2025-08-27 | 528 | +2.9 | +2.1 | +5.2 |
| 2025-08-28 | 1709 | +0.9 | +2.6 | −2.8 |
| 2025-08-29 | 161 | +3.3 | +1.7 | +4.8 |
| 2025-09-01 | 1006 | +0.6 | −0.7 | −7.8 |
| 2025-09-02 | 1062 | +4.3 | +2.6 | −3.8 |
| 2025-09-04 | 177 | −4.1 | −6.8 | −11.7 |
| 2025-09-05 | 821 | +3.8 | +1.6 | +16.8 |
| 2025-09-06 | 79 | −1.7 | −1.7 | +40.5 |
| 2025-09-07 | 1300 | +0.8 | +0.7 | −28.3 |
| 2025-09-08 | 799 | +8.7 | +8.5 | −0.3 |
| 2025-09-10 | 422 | +6.2 | +1.8 | +11.1 |
| 2025-09-12 | 522 | +15.7 | +18.8 | +1.4 |
| 2025-09-17 | 415 | +0.3 | −0.3 | −0.2 |
| 2025-09-22 | 156 | +4.7 | +4.1 | 0.0 |
| **Date-equal mean** | **—** | **+1.0** | **+0.9** | **−4.3** |

Supplementary Table D1. Inference latency and throughput of the vision and fused (concat) models on the target workstation (AMP = automatic mixed precision; throughput in leaves per second).

| Configuration | Vision latency | Concat latency | Weight-branch overhead | Throughput (vision / concat) |
| --- | --- | --- | --- | --- |
| GPU, batch = 1, AMP on | 9.00 ms | 9.25 ms | +0.24 ms | 111 / 108 leaves/s |
| GPU, batch = 1, AMP off | 7.96 ms | 8.57 ms | +0.61 ms | 126 / 117 leaves/s |
| GPU, batch = 32, AMP on | 68.86 ms | 69.39 ms | +0.53 ms | 465 / 461 leaves/s |
| GPU, batch = 32, AMP off | 114.06 ms | 113.75 ms | −0.31 ms | 281 / 281 leaves/s |
| CPU, batch = 1 | 204.1 ms | — | — | 4.9 leaves/s |

**Supplementary Table E1. Notation used in the Model section (Section 3.3).**

| **Symbol** | **Dim.** | **Meaning** |
| --- | --- | --- |
| x | 448 × 224 × 3 | cropped conveyor-belt RGB image |
| w | R | per-leaf in-line weight (g) |
| w_z | R | w standardized with training-fold mean/std |
| f | R^{2048} | image feature after GAP (ResNet-50 layer4) |
| h_w | R^{64} | weight embedding (2-layer MLP, ReLU) |
| v, q | R^{256} | projected image / weight representations (single Linear + ReLU) |
| g | (0,1) | scalar gate, g = σ(Linear([v;q])) |
| z | R^{256} | fused representation g·v + (1−g)·q |
| p_v, p_w | Δ^{8} | class-probability vectors (vision / weight) |
| y | {1..9} | grade label |
| d | {34 dates} | acquisition date (batch unit) |

**Supplementary Table E2. Specification of every learnable block in the fusion architectures.**

| **Block** | **Specification** |
| --- | --- |
| Image backbone | ResNet-50 (ImageNet); conv1–layer3 frozen, layer4 fine-tuned; GAP → 2048-D |
| Weight MLP | Linear(1→64) + ReLU + Linear(64→64) + ReLU |
| v projection | Linear(2048→256) + ReLU |
| q projection | Linear(64→256) + ReLU |
| Gate | Linear(512→1) + sigmoid |
| Concat fusion | Linear(2112→256) + ReLU |
| Classifier heads | single Linear(·→9) |
| Optimizer | AdamW, lr 1e-4 (backbone) / 1e-3 (heads), wd 1e-4, cosine + 1-epoch warm-up, label smoothing 0.05, EMA 0.995, AMP |
