# Supplementary material for: Fusing conveyor-belt images with existing in-line weight records for flue-cured tobacco leaf grading: a strict date-held-out evaluation
Source: Front Plant Sci. 2026 Oct 26;17:1970460. doi: 10.3389/fpls.2026.1970460 (PMC13745679; doi:10.3389/fpls.2026.1970460)
Supplement: Supplementary file 2 [file Table1.docx]

Supplementary Material

# 1 Supplementary Tables

Table A2. Complete grade × acquisition-date composition matrix (sample counts) for the 34 acquisition dates and 11 grades of the final paired image–weight dataset (N = 25,325). The last row gives per-date totals; the last column gives per-grade totals.

| **Date** | **B1F** | **B2F** | **B3F** | **C1F** | **C2F** | **C3F** | **C4F** | **C3L** | **X2F** | **X3F** | **X4F** | **Total** |
| --- | --- | --- | --- | --- | --- | --- | --- | --- | --- | --- | --- | --- |
| 2025-07-02 | 0 | 0 | 0 | 0 | 0 | 175 | 0 | 119 | 162 | 0 | 0 | 456 |
| 2025-07-03 | 0 | 0 | 0 | 0 | 0 | 0 | 0 | 0 | 176 | 0 | 0 | 176 |
| 2025-07-18 | 0 | 0 | 0 | 0 | 0 | 0 | 0 | 0 | 492 | 0 | 0 | 492 |
| 2025-07-19 | 0 | 0 | 0 | 0 | 0 | 419 | 0 | 0 | 0 | 0 | 0 | 419 |
| 2025-07-21 | 0 | 0 | 0 | 0 | 0 | 119 | 20 | 16 | 101 | 0 | 0 | 256 |
| 2025-07-23 | 0 | 0 | 0 | 0 | 294 | 0 | 294 | 233 | 0 | 0 | 0 | 821 |
| 2025-07-24 | 0 | 0 | 0 | 0 | 280 | 0 | 0 | 88 | 0 | 0 | 0 | 368 |
| 2025-07-30 | 0 | 0 | 0 | 0 | 0 | 0 | 0 | 0 | 227 | 0 | 0 | 227 |
| 2025-07-31 | 0 | 0 | 0 | 0 | 0 | 0 | 73 | 0 | 0 | 0 | 0 | 73 |
| 2025-08-01 | 0 | 9 | 4 | 0 | 210 | 197 | 37 | 177 | 44 | 346 | 0 | 1024 |
| 2025-08-02 | 0 | 0 | 0 | 0 | 50 | 231 | 137 | 315 | 0 | 0 | 129 | 862 |
| 2025-08-07 | 0 | 0 | 0 | 0 | 9 | 123 | 0 | 6 | 176 | 0 | 0 | 314 |
| 2025-08-08 | 0 | 0 | 0 | 0 | 57 | 41 | 77 | 0 | 0 | 0 | 0 | 175 |
| 2025-08-10 | 0 | 0 | 0 | 0 | 70 | 0 | 0 | 0 | 0 | 0 | 0 | 70 |
| 2025-08-12 | 0 | 0 | 10 | 11 | 0 | 16 | 59 | 0 | 0 | 0 | 0 | 96 |
| 2025-08-18 | 152 | 365 | 663 | 666 | 611 | 581 | 302 | 120 | 557 | 0 | 0 | 4017 |
| 2025-08-19 | 0 | 0 | 0 | 4 | 350 | 1396 | 498 | 0 | 493 | 0 | 0 | 2741 |
| 2025-08-22 | 0 | 0 | 0 | 7 | 139 | 269 | 0 | 176 | 0 | 0 | 578 | 1169 |
| 2025-08-24 | 0 | 0 | 0 | 0 | 0 | 795 | 439 | 59 | 645 | 0 | 0 | 1938 |
| 2025-08-26 | 0 | 0 | 0 | 0 | 0 | 221 | 0 | 75 | 178 | 0 | 0 | 474 |
| 2025-08-27 | 0 | 0 | 0 | 0 | 217 | 98 | 86 | 14 | 113 | 0 | 0 | 528 |
| 2025-08-28 | 0 | 0 | 0 | 357 | 279 | 620 | 452 | 1 | 0 | 0 | 0 | 1709 |
| 2025-08-29 | 0 | 0 | 0 | 0 | 161 | 0 | 0 | 0 | 0 | 0 | 0 | 161 |
| 2025-09-01 | 169 | 184 | 226 | 361 | 66 | 0 | 0 | 0 | 0 | 0 | 0 | 1006 |
| 2025-09-02 | 280 | 329 | 296 | 111 | 46 | 0 | 0 | 0 | 0 | 0 | 0 | 1062 |
| 2025-09-04 | 177 | 0 | 0 | 0 | 0 | 0 | 0 | 0 | 0 | 0 | 0 | 177 |
| 2025-09-05 | 265 | 360 | 196 | 0 | 0 | 0 | 0 | 0 | 0 | 0 | 0 | 821 |
| 2025-09-06 | 0 | 0 | 0 | 0 | 0 | 49 | 0 | 13 | 17 | 0 | 0 | 79 |
| 2025-09-07 | 440 | 426 | 434 | 0 | 0 | 0 | 0 | 0 | 0 | 0 | 0 | 1300 |
| 2025-09-08 | 114 | 233 | 252 | 200 | 0 | 0 | 0 | 0 | 0 | 0 | 0 | 799 |
| 2025-09-10 | 114 | 158 | 40 | 110 | 0 | 0 | 0 | 0 | 0 | 0 | 0 | 422 |
| 2025-09-12 | 173 | 196 | 153 | 0 | 0 | 0 | 0 | 0 | 0 | 0 | 0 | 522 |
| 2025-09-17 | 0 | 0 | 0 | 0 | 0 | 415 | 0 | 0 | 0 | 0 | 0 | 415 |
| 2025-09-22 | 54 | 25 | 15 | 0 | 62 | 0 | 0 | 0 | 0 | 0 | 0 | 156 |
| **Total** | **1938** | **2285** | **2289** | **1827** | **2901** | **5765** | **2474** | **1412** | **3381** | **346** | **707** | **25325** |
